# Fostering cardiovascular health at work – case study from Senegal

**DOI:** 10.1186/s12889-021-11109-9

**Published:** 2021-06-10

**Authors:** Ida Ndione, Ann Aerts, Asha Barshilia, Johannes Boch, Sarah Des Rosiers, Jose M. E. Ferrer, Jasmina Saric, Karim Seck, Bernard N. Sene, Peter Steinmann, Lakshmi Venkitachalam, Jason T. Shellaby

**Affiliations:** 1 PATH, Dakar-Fann, Dakar, Senegal; 2 Novartis Foundation, Basel, Switzerland; 3 American Heart Association, Dallas, Texas USA; 4 Swiss Tropical and Public Health Institute, Basel, Switzerland; 5 University of Basel, Basel, Switzerland

**Keywords:** Workplace health and wellness, Cardiovascular health, Non-communicable disease, NCD, Hypertension, Africa, Better Hearts Better Cities

## Abstract

**Background:**

Of the 15 million annual premature deaths from non-communicable diseases (NCDs), 85% occur in low- and middle-income countries (LMICs). Affecting individuals in the prime of their lives, NCDs impose severe economic damage to economies and businesses, owing to the high mortality and morbidity within the workforce. The Novartis Foundation urban health initiative, Better Hearts Better Cities, was designed to improve cardiovascular health in Dakar, Senegal through a combination of interventions including a workplace health program. In this study, we describe the labor policy environment in Senegal and the outcomes of a Novartis Foundation-supported multisector workplace health coalition bringing together volunteering private companies.

**Methods:**

A mixed method design was applied between April 2018 and February 2020 to evaluate the workplace health program as a case study. Qualitative methods included a desk review of documents relevant to the Senegalese employment context and work environment and in-depth interviews with eight key informants including human resource representatives and physicians working in the participating companies. Quantitative methods involved an analysis of workplace health program indicators, including data on diagnosis, treatment and control of hypertension in employees, provided by the coalition companies, and a cost estimate of NCD-related ill-health as compared to the investment needed for hypertension screening and awareness raising events.

**Results:**

Senegal has a legal and regulatory system that ensures employee protection, supports social security benefits, and promotes health and hygiene in companies. The Dakar Workplace Health Coalition comprised 18 companies, with a range of staff between 300 and 4′220, covering 36′268 employees in total. Interviews suggested that the main enablers for workplace program success were strong leadership support within the company and a central coordination mechanism for the program. The main barrier to monitor progress and outcomes was the reluctance of companies to share data. Four companies provided aggregated anonymized cohort data, documenting a total of 21′392 hypertension screenings and an increasing trend in blood pressure control (from 34% in Q4 2018 to 39% in Q2 2019) in employees who received antihypertensive treatment.

**Conclusion:**

Evidence on workplace health and wellness programs in Africa is scarce. This study highlights how private sector companies can play a significant role in improving cardiovascular population health in LMICs.

**Supplementary Information:**

The online version contains supplementary material available at 10.1186/s12889-021-11109-9.

## Background

Each year, an estimated 15 million people between 30 and 69 years are dying prematurely from non-communicable diseases (NCDs), and 85% of those deaths occur in low- and middle-income countries (LMICs) [1]. The highest probability of premature mortality (22%) is found in Africa, followed by 16% in the Western Pacific region and 15% in the Americas [1].

The epidemiological trend in Senegal is representative for that in other West African countries, where infectious disease burden is being overtaken by NCDs, both in terms of health and economic impact [1, 2]. The most recent report from the World Health Organization (WHO) estimated that, in 2016, NCDs accounted for 42% of the overall mortality in Senegal – with 17% of all deaths caused by cardiovascular disease (CVD) [1]. Hypertension, the prime risk factor for CVD, was found highly prevalent in Senegal’s 2015 STEPS survey, with almost a third (29.8%) of the 18–69 year old population suffering from the condition [3]. Less than half of the patients with high blood pressure (BP) (46%) were aware of their condition, while only 17% reported taking antihypertensive therapy and BP control was reached in a mere 8% of all patients (unpublished data).

Better Hearts Better Cities is a global urban health initiative, launched in 2017 by the Novartis Foundation. It aims to improve cardiovascular population health in urban LMIC settings, in a sustainable way at scale. The initiative was implemented in Ulaanbaatar, Mongolia; Dakar, Senegal; and São Paulo, Brazil and deploys an innovative multidisciplinary, multisector approach to address hypertension and its underlying determinants [4]. Recognizing the need to bring health and care closer to where people live and work, one of the initiative’s goals was to address CVD in the workplace by accelerating detection and improving hypertension management in employees, as well as improving cardiovascular health literacy.

The role of workplace environments in the prevention and control of NCDs has been emphasized by the international community since 2012/2013, when the United Nations and WHO called for a ‘whole-of-government, whole of-society’ approach to NCDs, including the creation of an ‘enabling environment for healthy behaviors among workers’ [5, 6]. Workplace health programs have indeed demonstrated important benefits for businesses, such as decreased absenteeism [7–9], increased likelihood of smoking cessation and decreased systolic BP in employees. Yet, such programs remain scarce in LMICs as national NCD policies and plans often fail to account for the workplace as an essential contributor to the prevention and management of NCDs [10, 11]. Across Africa, only 1% of employees are estimated to have access to workplace health programs, which is also reflected in the scarcity of literature on NCD workplace health programs on the continent [12–14]. However, some good examples can be found on the continent, such as the Employee Wellbeing Programs in Ghana, supported by the German Federal Ministry for Economic Cooperation and Development. This program managed to increase BP control rates in employees by 18% over 4 years of implementation [15]. A 2017 report published by the NCD Alliance and the Novartis Foundation provides a comprehensive overview on progress, challenges, and opportunities in workplace health [11]. While underlining the necessity to provide tools and support to implement workplace programs, the report demonstrates how workplace interventions can be embedded within local health system strategies. The report was the basis for establishing the workplace health program in Dakar within the Better Hearts Better Cities initiative [11].

Since April 2013, Senegal has an NCD Unit in its Ministry of Health and Social Action (MOHSA). Its special office focusing on CVD has been supporting the implementation of Better Hearts Better Cities and its workplace program. The National Health Development Plan 2009–2018, already specified that the fight against NCDs demands intra-, inter-, and multi-sectoral collaboration, including with private companies. In 2018, the Minister of Labor emphasized that ‘Employers will have to demonstrate a greater awareness of their obligations regarding health and safety at work and for workers’. According to recent estimates, the service sector accounts for 57% of total national employment, the agriculture sector for 29%, and industry for 14% [16]. Only about 8% of the working age population is formally employed, based on modelled data from the National Agency of Statistics and Demography of Senegal [17]. Gender disparities remain high with women’s access to education and participation in the labor market estimated at 35% in 2018 [17]. Labor market participation by age group is highest in those aged 45–55 (62%) and lowest in the age group of 15–25 (29%) years [18]. In Dakar, most employers in the formal sector offer health insurance to their employees and families, an added incentive to address health in the workforce. Although targeting the formal and/or private sector may not initially reach the majority of employees in Senegal, establishing a solid concept for workplace health in a business environment can help the informal sector to adapt it to their needs, as a next step. Moreover, given that the largest part of employees in the formal sector are over 45, workplace health schemes such as this one, can capture a high proportion of the people most at-risk for NCDs.

This study aimed to highlight the opportunity for Senegal and other countries with an equally enabling political and policy environment, to further strengthen effective, locally adapted, and sustainable workplace health programs. We conducted an initial analysis of the workplace health environment and assessed the role private sector companies can play to enhance awareness, prevention and management of hypertension in Dakar. We describe opportunities, challenges, and lessons learned from this workplace health program, supported by the Better Hearts Better Cities teams.

## Methods

### Study design

We applied a mixed method design to describe the labor policy environment in Senegal and the outcomes of a multisector coalition of volunteering private companies in Dakar that was supported by Novartis Foundation. The PATH Dakar office acted as implementation partner, assuring local coordination. A detailed description of the qualitative and quantitative methods is given below. Findings were triangulated with orally reported or documented observations by the authors. Approval to generate routine data in Dakar within the framework of the Better Hearts Better Cities initiative was given by the Comité National d’Ethique pour la Recherche en Santé (Protocol SEN18/79) and a collaboration agreement was in place between MOHSA and the Novartis Foundation. All methods were carried out in accordance with the relevant national guidelines and regulations.

### Document review

A desk review of reports, presentations, national strategic plans, regulatory texts, and legal files covering the Senegalese employment context and work environment was conducted by a team of two authors (IN, BS). Initially, key documents and organizations were identified in consultation with the PATH Human Resources office. To identify further relevant documents, representatives of those organizations were approached in a second phase; they included the Labor and Social Security Inspector (Inspecteur du Travail et de la Sécurité Sociale) at the Ministry of Labor and representatives of the Social Security Fund and the International Labor Organization. The Social Security Code and International Labor Organization standards [19, 20] were identified as key documents and a thematic analysis was applied to extract anything relating to “health” and the responsibility of companies with regards to the health of their employees.

### Interviews

Interviews with company stakeholders were conducted including five occupational physicians and two company leadership members (one Human Resources Director and one Health, Safety and Environment Manager), as well as the Occupational Safety Manager of the National Council of Employers (Conseil National du Patronat). Interviewees were selected by a purposive sampling approach. The impact and enabling or challenging factors for workplace health were assessed using a standard semi-structured questionnaire, also exploring the role of employees in workplace health programs. The conceptual template of the questionnaire was informed by the Pillars of Workplace Health used by the American Heart Association’s CEO Roundtable to promote evidence-based approaches to workplace health [21]. Questions were structured around i) impact of the Better Hearts Better Cities workplace health program in the company; ii) success factors, barriers and opportunities for implementing the program; iii) employee engagement; and iv) experience for the company as a member of the workplace health coalition. This included an assessment of the behavior changes and lifestyle choices of employees, as well as their reactions to the workplace program, as perceived by the physicians and company leadership. While the questionnaire was emailed to interviewees in advance, the in-person interviews were conducted by a PATH researcher, lasted between 1 and 2 hours, and were recorded for transcription and data analysis. Data confidentiality was ensured at each step and every participant provided written informed consent prior to the interview. The information from qualitative interviews was further supplemented with data provided by 18 companies, using a structured questionnaire, on key company characteristics including employee size, number covered by workplace intervention and presence of pre-existing workplace wellness programs. A deductive analysis of interview transcripts was conducted, guided by the questions and key areas of interest, i.e., strengths, weaknesses, and opportunities.

### Quantitative data and data analysis

Quarterly aggregated de-identified hypertension cascade data were collected from participating companies between April 2018 and March 2019. Based on the lack of a denominator for screening (number of total employees per screening) and the lack of cumulative data for persons analyzed and persons treated, both diagnosed and treated were represented as a proportion of screened. For control, treatment was used as denominator, allowing for calculation of proportions of patients controlled among those treated and their corresponding 95% confidence intervals. The temporal trend in the numbers of employees controlled for hypertension, was analyzed using the chi-squared test for trend and a binomial regression on the aggregate numbers, with time as the only explanatory variable. Crude estimates of costs of NCD-related disability and absenteeism were calculated based on published estimates of average salary and estimated employee productivity, as well as obligations following absence and disability (as defined in the Social Security Code and International Labor Organization standards) [19, 20], and compared to the investment needed for hypertension screening and awareness raising events.

### Dakar Workplace Health Coalition

While planning Better Hearts Better Cities, the need for a workplace health coalition between private sector companies was identified. The Dakar Workplace Health Coalition was set up to facilitate private sector engagement in tackling CVD, support companies to design activities addressing hypertension and its underlying determinants at the workplace, and provide them with a space to discuss feasibility and challenges of running workplace health programs.

The coalition’s objectives were defined as i) establishing a health committee to address hypertension; ii) creating an enabling environment for optimizing healthy food options at the workplace; iii) conducting physical education sessions within the workplace, while encouraging employees to continue exercising; and iv) creating a smoke-free environment (Decree n°2016–1008 of July 26, 2016; implementing Law n° 2014–14 of March 28, 2014 on the manufacturing, packaging, labelling, sale and use of tobacco) [22]. Initially, only companies with prior experience in workplace health were admitted to the coalition, while other membership prerequisites were a strong support from company leadership for workplace health, as well as the availability of occupational health personnel (physician or nurse). Member companies were invited to discuss and explore how to best engage and collaborate, with the Better Hearts Better Cities team acting as a facilitator and organizing the launch of the coalition. There were no monetary incentives for participating companies, but companies benefited from training, screening activities and participation in roundtables discussions.

Members signed the Charter of the Coalition Agreement (Charte d’Engagement), committing to implement at least twice a year a set of interventions promoting health education on hypertension and its risk factors, and to facilitate early detection and adequate management of high BP within the employee populations. The main interventions were i) offering health education on high BP on World Hypertension Day; ii) promoting a balanced diet; ii) encouraging physical activity; iv) reducing stress and tobacco use; and v) providing information on healthy lifestyle. Success of the interventions was assessed by the number and proportion of employees covered by workplace health activities and the hypertension cascade data in employees (main outcomes variables). The number of companies that requested to organize similar activities at their other worksites, was also considered an indicator of success, as was the positive feedback from medical, administrative and leadership staff.

At the initial creation of the Dakar Workplace Health Coalition in November 2017, invitation letters were sent to over 40 companies. These were selected out of an existing network from the implementing partner PATH. As part of a proactive outreach, larger companies in Dakar and those known to have organized workplace health initiatives previously, were approached directly. Four companies responded positively to the invitation and formed the core founders of the coalition. Later on, initial admission criteria were simplified and again several companies were invited to join, resulting two years later in 18 company coalition members representing over 36′000 employees, with the smallest companies counting 300 employees and the largest more than 4000 (Table 1). These included four multinational and 14 national companies, with only two (multinational) companies reporting to have existing workplace health programs at the outset. The diversity of the members (i.e. companies active in construction, food and beverages and transport), employees with different income and education levels were included. All companies offered access to medical services, either through an on-site physician or outsourced services, and participation in workplace health activities was allowed during working hours. Only four of the member companies (one multinational and three national ones) were willing to share employee health data.

**Table 1:** Companies participating in the Better Hearts Better Cities Dakar Workplace Health Coalition

Company	Number of employees	Type of company	Pre-existing workplace health program (yes/no)	Approximate number of employees covered by workplace health activities	Implementing workplace health in any other site than Dakar (yes/no)	Collecting employee data on hypertension (yes/no)	Companies sharing employee health data (yes/no)
C1	1900	Multinational	Yes	1000	No	Yes	Yes
C2	4000	National	No	800	No	Yes	Yes
C3	4220	National	No	300	No	No	No
C4	500	National	No	70	No	No	No
C5	1000	National	No	150	No	No	No
C6	2500	National	No	300	No	No	No
C7	330	Multinational	Yes	50	No	No	No
C8	1400	National	No		No	No	No
C9	1650	National	No	300	No	Yes	Yes
C10	2000	Multinational	No	300	No	Yes	No
C11	3268	National	No		No	No	No
C12	3500	National	No	500	No	Yes	Yes
C13	300	National	No		No	No	No
C14	1200	National	No		No	No	No
C15	3000	National	No		No	No	No
C16	1200	National	No		No	No	No
C17	1500	National	No		No	No	No
C18	2800	Multinational	No		No	No	No

Limited understanding of the economic benefit offered by health at the workplace, may have kept local companies in Dakar from engaging in the coalition. Other companies may have been reluctant to join following a previously failed attempt to set up a similar workplace health coalition. Given that the lack of concrete support for organizing workplace health activities was cited as a major reason for that previous failure, the Better Hearts Better Cities team decided to take a different approach and provide true operational and pragmatic guidance.

Besides representatives from the company leadership teams, human resource departments and occupational health providers, the coalition included officials from the Private Medicine and NCD Division at the MOHSA. All activities of the initiative were conducted with implementing partners (NGOs) and local consultants. The coalition also engaged with the National Council of Employers, the Social Security Fund, the Federation of Insurance Companies and the Association of Occupational Physicians. Meetings offered member companies the opportunity to i) review the business case of prevention and addressing hypertension in the workplace; ii) share information about existing workplace health programs; iii) understand the importance of such programs; and iv) brainstorm about new ways to address cardiovascular risk factors, at the workplace. As companies expressed the need for communication materials on hypertension, Better Hearts Better Cities also supported the creation of such tools. Workplace health interventions included screening and awareness campaigns, health provider training on hypertension diagnosis and treatment, collection of hypertension cascade data (employees screened, diagnosed, treated and controlled for hypertension), and information sharing for outreach.

## Results

### Legal and regulatory situation in Senegal

The desk review of documents related to the Senegalese employment and work environment revealed that the country has ratified several conventions in the field of social security and employee protection with the International Labor Organization (ILO). Senegal also has a legal and regulatory system to preserve social security, hygiene and health of employees (SSHHE) [19, 20, 22, 23], including the Social Security Fund and Labor Inspectorate. Senegal’s social security scheme for salaried workers covers most risks, except unemployment. The government included health policies to strengthen SSHHE in the National Health Development Plans and in the Strategic Plan against NCDs. Compulsory health insurance in Senegal is governed by legal instruments [20], making affiliation mandatory for employers, although the benefit package that health insurers offer may vary, and prevention of NCDs is often not included.

### Insight from company stakeholder interviews

Interviews with the selected company representatives revealed that workplace programs were perceived as beneficial, especially with regard to training and health education and offering more training and educational material was widely recommended by both physicians and leadership members. Company physicians also mentioned an increased understanding of the NCD burden amongst their employees and recognized a paradigm shift, with patients taking more responsibility in the management of their own health, being more active, and compliant with health recommendations. Also patients’ feedback was mainly positive. Company leadership felt that nutritional education offered through the Better Hearts Better Cities workplace program had increased employees awareness about the benefits of healthy lifestyles (Insights summary in Table 2).

**Table 2:** Insights from company representative interviews

**Occupational physicians**	
• Support of company leadership to enable workplace health is essential ° The training offered by Better Hearts Better Cities was highly valued, not only for its materials, tools and themes covered, but also for the peer-to-peer exchanges • The workplace health program could benefit from more Better Hearts Better Cities training and materials, and in particular: ° For occupational physicians: more training on the integrated management of diabetes and metabolic disease, as well as on the prevention and management of overall cardiovascular risk. Simple decision support systems and training on the use of devices for patient follow up (e.g. electrocardiogram and ambulatory blood pressure monitoring) was also found useful ° For leadership members: on all NCD risk factors (e.g. including stress) ° For employees: on healthier lifestyles and ways to reduce risk • Regularity and continuity of workplace health activities is key • The most successful strategies for engaging employees in their own health were reported to be the health education sessions (individual or collective), the regular blood pressure checks and/or medical visits ***Management and administrative leadership members***	
• Including employees in designing workplace health programs is essential to nurture ownership and expand motivation to their peers • Occupational physicians are perceived as health champions who should have good communication skills, capable of relaying the right information to employees • Materials should also exist in Wolof, the local language in Senegal • More guidance for progress and outcome assessments would be appreciated, especially using a standardized set of indicators that can be compared over time and between companies • A solid action plan with a clear vision for sustainable impact is critical	

### Workplace health activities

#### Screening and awareness sessions

Hypertension screening and educational sessions were often designed as combined events by the participating companies and included, at times, a survey about the employees’ lifestyle. Prevention of NCDs and healthy nutrition were focus themes for the awareness sessions organized by the companies. Nutritionists were consultated either for the design of the campaign or for their direct involvement in discussion with employees. Such sessions highlighted the link between unhealthy lifestyles and the risk of developing hypertension, diabetes and CVD. Employees seemed to have understood the risk of high BP, but were unaware that lifestyles such as limited physical activity or diets high in salt, fat or sugar, could influence it. This education increased health literacy and its combination with regular BP measurements was considered most successful to engage employees in the management of their own health. Other activities, such as organized walks, were also leveraged, often attracting a considerable number of participants (up to 3000 per event) and reportedly creating a snowball effect on the community at large, as e.g. family members frequently joined.

#### Training health professionals

Workplace physicians and nurses were trained on the prevention and management of hypertension and CVD, with a similar curriculum as the one Better Hearts Better Cities rolled out for primary healthcare professionals throughout the city, albeit tailored to the specific needs of occupational health providers. Training was organized in collaboration with the National Association of Occupational Physicians and the NCD Division of the MOHSA and the Sengalese Society of Cardiology (Société Sénégalaise de Cardiologie; SOSECAR). Participating health staff reportedly appreciated the interaction and exchange with their peers, while also expressing the need for more educational materials (see interview section for full details).

#### Data collection on the hypertension cascade

**Fig. 1:**
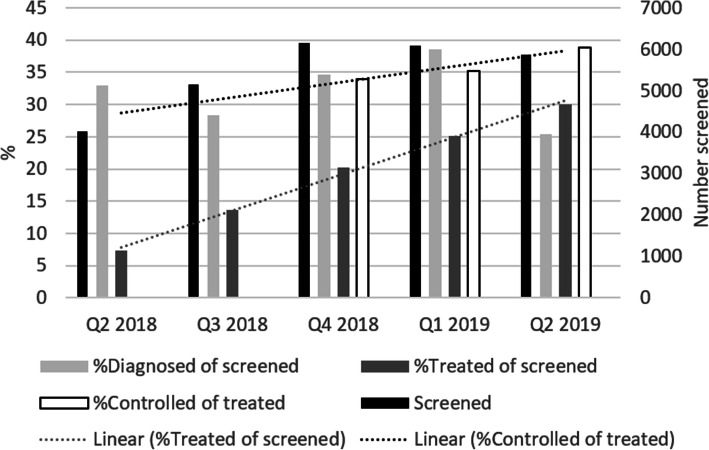
Hypertension cascade data pooled by quarter


Coalition members committed to share quarterly results of their workplace health programs, on the number of employees screened, diagnosed, treated and controlled for hypertension. Data were collected in aggregate and anonymized form between April 2018 and March 2019 (Supplementary Material Data). Only four out of the 18 coalition companies ultimately provided the data (Table 1), as companies expressed a fear that sharing data could indicate poor employee health and potentially harm their image. Within the data collection period, an increase was observed in employees screened for hypertension (Fig. 1). Between Q2 2018 and Q2 2019, 21′392 hypertension screenings were conducted and patients treated amongst those screened increased from 7% to 30%. BP control and referral data were not consistently available. However, for those time points where a full set of patients with controlled BP was available, proportions of people with controlled BP amongst those treated and 95% confidence intervals were computed as follows: 0.34 (0.313–0.366) for Q4 2018; 0.35 (0.329–0.377) for Q1 2019 and 0.39 (0.365–0.411) for Q2 2019; *p*-value for trend = 0.005.

**Additional file 2.**


#### Information sharing

Educational material was shared with all companies invited to join the coalition, regardless of their membership. Non-member companies were also invited to the coalition meetings and events. Materials supporting the coalition’s governance and operations are listed in Table 3 and available as Supplementary Material (except for a Flyer and a second Kakemono that contained images unsuited to open access publication; they can be provided, however, by the authors upon request). During World Hypertension Day and World Diabetes Day, Better Hearts Better Cities offered support to companies in Dakar to increase their outreach and awareness campaigns on hypertension and its risk for cardiovascular complications. For the 2018 World Hypertension Day Celebration for instance, two days of screening were combined with health education sessions on hypertension and its risk factors. Of the 593 persons who had their BP measured during that event, 136 had high BP and 80 had their diagnosis confirmed by the company physician.

**Table 3:** Materials and products supporting the Workplace Health Coalition governance and operations

Item	Description
***Governance support***	
Charter for Coalition Agreement	Charter of commitment to the fight against hypertension and to promote healthy lifestyles
Training Certification	For medical professionals participating in the training on the treatment of hypertension
LEADERS Framework	Support for business strategies for workplace health
***Operational material***	
Video clip^a^	Educational clip on hypertension risk factors, symptoms and prevention in French, with/without English subtitle and in Wolof
Flyer	Educational flyer on hypertension risk factors, complications, prevention and the role of the workplace
Notebook	Promotional notebook containing information on non-communicable diseases, the Better Hearts Better Cities initiative and its workplace program, diabetes and hypertension, prevention and risk factors
Screening cards	For documenting systolic and diastolic blood pressure, body weight and height and capillary glycaemia
Kakemono	Educational flyer on hypertension risk factors and prevention
T-shirt models	Featuring key-messages of hypertension prevention

^a^The video clip was produced by PATH and is owned by the Better Hearts Better Cities initiative

In 2019, a working group including members of NCD Division, the National Health Education Service (Service National de l’Education et de l’Information pour la Santé; SNEIPS) of the MOHSA, and the occupational physicians within the coalition, was set up to design health education material for employees and their families. As such, a local cartoon was developed for instance (in French with English subtitles and in Wolof), to increase health literacy about hypertension, its risk factors and complications, including information on how to best avoid or control it (Supplementary Material). The format and different versions of the cartoon made large scale dissemination possible via national television, health facilities, schools, public transportation and through the participating coalition companies.

### Cost estimate for NCD prevention activities versus ill-health

The screening and sensitization efforts at an average company in Dakar with 200 employees would require approximately 650 Euro worth of sphygmomanometers, glucometers, scales and height measuring equipment, plus approximately EUR 3 per employee for medical consumables (Table 4). One day of absenteeism at an average company in Dakar, would cause the company approximately EUR 21 in direct costs for an employee with a EUR 460 monthly salary, and a 0.09% loss in monthly turnover as indirect costs (Table 5). If the same employee would suffer disability, direct costs for the company would amount to EUR 479 per month (for an average of 6 months), in addition to the expenses for recruiting and training a replacement. The 6 months average was based on estimates communicated by the physicians at two of the coalition companies, and referring to the event of stroke and resulting disability.

**Table 4:** Estimated cost of organizing screening and sensitization events at a company in Dakar with 200 participants

Item	Quantity	Local price per unit (€)	Total (€)	Cost per beneficiary (€)
***Basic investment***				
Omron blood pressure monitor	5	61.2	305.9	NA
Scales	2	30.6	61.2	NA
Height chart	2	22.9	45.9	NA
Measuring tape	5	2.3	11.5	NA
Glucometer	5	45.9	229.4	NA
Total			653.9	
***Investment for 200 beneficiaries***				
ACCU-CHEK strip/50	10 boxes of 50	16.8	168.2	0.8
Urinary strip Siemens Multistix 10 SG/100	10	19.1	191.2	1.0
Gloves	5 boxes of 10	6.1	30.6	0.2
Sterile self-injecting syringe ACCU-CHEK 200	5 boxes	19.9	99.4	0.5
Alcogel	10 bottles	3.8	38.2	0.2
Compresses	10 boxes	5.4	53.5	0.3
Cotton	3	1.5	4.6	0.0
Total technical material			1893.4	3.0

**Table 5:** Example of costs pertaining to ill-health of company employees in Dakar

Situation in a company with 50 employees	Manifestation	Direct costs	Indirect costs
Absenteeism of an employee who earns € 458.8 per month	Assumption of one day of absence per month € 458.8/ 173 h * 8 h	€ 21.2 per month per day of absence	Overall monthly production of the company reduced by 0.09% for each day of absence of an employee
Disability following a complication of an employee earning € 458.8 per month	During a re-education period of 6 months, the employer is required to pay the salary under penalty of a fine and imprisonment (article L279 labor code)	€ 2875.2 for 6 months This cost can extend to a year or more depending on regulatory provisions (article 19 and 20 of the Collective Trade Agreement)	Temporary replacement costs for a new recruit with the same monthly salary of € 458.8 to maintain production. The costs of rehabilitation or functional rehabilitation are the responsibility of the Social Security Code (Article 58)

## Discussion

### Major findings

Senegal has a legal and regulatory system that ensures formal employee protection, supports social security benefits, and promotes health and hygiene in companies. In this context, the Dakar Workplace Health Coalition, comprising 18 medium-sized and large companies, succeeded in integrating hypertension screening, diagnosis and processes leading to treatment in employees and raised the proportion of employees with controlled hypertension. Between April 2018 and March 2019, 21,392 hypertension screenings were conducted. Although only a small data set was available, we could observe an increase in the proportion of treated hypertension patients who achieved blood pressure control, which is highly unlikely to be due to chance (*p* = 0.005). This should encourage companies in LMICs to pursue similar workplace health coalitions and approaches.

Comparing the costs of prevention at the workplace in this program, with those of occupational disease, clearly demonstrates an important financial incentive for businesses to integrate NCD prevention in the work environment. The Better Hearts Better Cities workplace health interventions were effective in raising health literacy on hypertension and its cardiovascular risk amongst employees, and in engaging the private sector, as well as civil society in workplace health. The approach created a positive exchange amongst companies and when companies organized activities including the media for instance, they attracted other companies to join the coalition. We observed that representatives from other companies who participated in the activities, would engage their leadership and human resource departments to also join the coalition’s efforts. Most importantly, while several companies replicated the activities at other worksites in Senegal, a national coalition is currently being established to continue the Better Hearts Better Cities workplace health initiative. This involves the Association of Company Physicians, the MOHSA’s Private Medicines Division and the Labor Department and should help assure long term sustainability of Senegal’s private sector engagement in workplace health.

Support by the company leadership, a central coordination mechanism, the integrity of the coalition and the strong multisector collaboration within Better Hearts Better Cities were considered key success factors for the workplace health program and its sustainability. Moreover, in-house rather than outsourced health service provision was considered important, while occupational physicians were perceived as champions for providing health education throughout the company. Some of these success drivers have already been identified in previous work, such as e.g. the perception in the NCD arena that only sustained multisector collaboration can lead to deep structural changes on the social, economic and political level, and achieve sustained behavioral change in the population [14, 24, 25]. The importance of leadership support and active involvement of senior company leadership was also previously reported in a workplace health study at a South African power plant [12]. There, leadership endorsement of the initiative enabled company-wide change, as were managers demonstrating personal commitment to behavior change. In Dakar, workplace health programs with defined, time-bound action plans and clearly defined roles and responsibilities were the most successful.

A lack of trust amongst member companies, on the other hand, was found to be a major challenge for the coalition resulting in limited data sharing and hesitation to discuss health related issues at the coalition meetings. Eventually, member companies saw the value of engaging with others, when realizing that the coalition also delivered support for organizing and implementing relevant workplace health activities. This enhanced engagement within the coalition ultimately drove a more positive and productive collaboration.

### Limitations

Companies participating in the coalition struggled with monitoring progress and outcomes of the workplace health activities. Although coalition members agreed to share quarterly aggregated hypertension cascade data, only a quarter of them did. In addition to a certain lack of trust, the fact that data were collected following different protocols and shared in aggregate form, significantly limited our scope of data analysis and consequently the value of our results. Data sharing in general and certainly in health is a complex undertaking, but potentially even more so between private companies, as highlighted here. We believe that building a standard monitoring system (ideally digitized) to generate evidence on improved health outcomes of employees, would benefit businesses [26]. Knowing that such a standardized system is operating and used by championing companies, may also reduce the reluctance for other companies to join and share data. To evaluate the impact of workplace health programs on employee health, data on health-related absenteeism should also be collected systematically, besides the hypertension cascade data. In Senegal, this information can be found in annual workplace health indicators that companies submit to the Social Security Fund, which would have the potential to provide companies with improved understanding of health-related productivity loss.

Another limiting factor of the program was the fact that it did not include employees in the business coalition at its conception and development phase. Their representation would certainly have contributed to a better understanding of the health needs in employee populations and could have refined the design of the workplace programs. Health and wellness programs in the workplace in India described how baseline employee surveys helped better understand employees’ needs and design workplace health programs accordingly [27].

Finally, there was a lack of process documentation, for the workplace program to become integrated into the national health system. As most companies in Dakar rely on private health providers for occupational health, it was difficult to link screening and newly diagnosed patients in the employee populations, to the local health system for follow up.

### Future directions

The WHO High Commission for NCDs states that every $ 1 invested in NCD interventions could give a return of $ 7 [28]. Many studies, mostly in high-income settings, have demonstrated that workplace health programs can reduce absenteeism, and medical costs [7–9, 29], while improving productivity [30]. Comparing the costs of prevention at the workplace with those of disease in the current study, clearly demonstrates the financial incentive for businesses to integrate NCD prevention in the work environment. Yet, very few programs that address employee health in a workplace environment in LMICs are known and fewer still are specific to NCDs [15, 31]. This is in spite of a legal and regulatory environment that is accommodating private sector health initiatives as the one we describe in Senegal. Of the 15 member states of the Economic Community of West African States (ECOWAS), Senegal is ranked 7th for its ILO Convention ratifications [32] and solid legal and regulatory systems are in place to protect employees and their families, by strengthening the SSHHE. However, there are a variety of Technical Conventions that leave space for improvement, i.e., only 31 of 178 have been ratified so far, including, e.g., the “Medical Examination of Young Persons” in the Industry environment.

It is unclear why only few employers in Senegal invest in workplace health programs despite considerable evidence of the impact of NCDs on business profitability. We hope that, by comparing the costs of workplace health programs with the costs of absenteeism and disability, the Dakar business coalition partners recognize the long-term economic value of workplace health programs and ensure their sustainability. Besides reducing absenteeism and disability related costs and improving employee wellbeing, companies investing in SSHHE can guarantee more stable and efficient productivity and operations and may be more likely to attract and retain employees. Ultimately, workplace health programs may also help stabilize insurance premiums for employers [33].

The experience of the Better Hearts Better Cities Dakar workplace health program reinforces the need for the LEADERS Framework for guidance and consistency [11], drawing on insights from several workplace health models from global health institutions. The framework recommends the development of action plans with measurable goals, activities and timelines for workplace health activities. Companies can then be rated in different categories, according to their performance in employees’ health and financial and organizational capabilities (Table 3, Supplementary Material). For this framework to thrive however, the integration of workplace health programs within local health systems and communities is essential. In addition, models for prevention and management of NCDs in the workplace that include all key players, have proven the most successful: only when private sector, public authorities and civil society work together and co-design the approach, will stakeholders across sectors be mobilized. Clear indicators to monitor progress and results, including health outcomes (e.g. health-related absenteeism) and company performance and productivity measures (e.g. health insurance premiums) can generate the much needed evidence of what works best in workplace health.

## Conclusion

This case study demonstrates that a relatively small investment from companies in workplace health can support employees in detecting and managing hypertension, the prime risk factor for CVD. Key enabling factors of the Better Hearts Better Cities workplace health program in Dakar were the formation of a business coalition to stimulate the launch and implementation of workplace health programs. The coalition provided support for the organization of health education and prevention activities, incentives and materials, and promoted peer influence amongst companies and employees. Ensuring high-level endorsement from company leadership, as well as strong multisector collaboration, were essential success drivers for workplace health programs. Despite some encouraging results, the lack of comprehensive data in our current study was a major challenge, hindering its potential to properly assess progress and impact. For sustainable replication of workplace health programs, employers and company leaders should commit to clear measurement of outcomes and impact of workplace health programs. The multisector urban health initiative, Better Hearts Better Cities, in Dakar underlines the significant opportunity for businesses to contribute to advancing cardiovascular population health. This documentation of the strategies and the long-term economic benefits complements the scarce evidence on NCD-oriented workplace health programs in the West African region and LMICs in general, and we hope that it may offer guidance to similar future programs.

## Supplementary Information

**Additional file 1.**

## Data Availability

The datasets used and/or analyzed during the current study are available as Supplementary Material.
