# Supplementary material for: Fostering cardiovascular health at work – case study from Senegal
Source: BMC Public Health. 2021 Jun 10;21:1108. doi: 10.1186/s12889-021-11109-9 (PMC8194249; doi:10.1186/s12889-021-11109-9)
Supplement: Supplementary file 2 — Additional file 1. [file 12889_2021_11109_MOESM1_ESM.zip › Coalition Charter Companies.pdf]

« BETTER HEARTS BETTER CITIES »

COALITION DES ENTREPRISES POUR LA LUTTE CONTRE L'HYPERTENSION ARTÉRIELLE ET LA PROMOTION DE SAINES HABITUDES DE VIE :

CHARTE D'ENGAGEMENT

DES EMPLOYÉS EN BONNE SANTÉ C'EST UNE ENTREPRISE EN BONNE SANTÉ

I- LES ENJEUX DE LA LUTTE CONTRE L'HYPERTENSION ARTÉRIELLE DANS LES ENTREPRISES

Les maladies non transmissibles (MNT) et l'hypertension artérielle (HTA) en particulier constituent une menace pour le développement et nécessitent des décisions de santé publique. Près de 30% des adultes sénégalais sont aujourd'hui concernés par l'Hypertension artérielle. Les conséquences de cette maladie sont potentiellement graves : accident vasculaire cérébrale (AVC) complication cardiovasculaires, insuffisance rénale… Face à cette situation, il est urgent d'agir, non seulement pour améliorer la prise en charge des personnes hypertendues, mais également d'agir en amont par la prévention de ses facteurs de risques très souvent liés à nos habitudes de vie (alimentation riche en graisse, sucre et sel, sédentarité, stress, consommation de tabac et alcool).

L'entreprise présente des avantages uniques pour parler de promotion de la santé et de bien-être. C'est là en effet que les travailleurs (qui constituent la majorité de notre cible) se rassemblent. Outre cette proximité physique, il y a la responsabilité des entreprises à offrir un lieu de travail sain. Par ailleurs, les études ainsi que l'expérience de nombreuses entreprises montrent qu'investir dans la prévention et la promotion de la santé permet d'économiser sur les coûts directs et indirects de la santé.

Le lieu de travail constitue un terrain privilégié pour sensibiliser une partie importante de la population que sont les salariés. Promouvoir la santé au travail est une manière positive pour les entreprises d'optimiser la gestion des ressources humaines et de prévenir les difficultés directement en lien avec l'état de santé des salariés. Quel chef d'entreprise n'est pas confronté à l'absentéisme lié aux arrêts de travail à répétition, à la nécessité de reclassement d'agents souffrant d'incapacités liés à une pathologie chronique, ou au besoin d'aménagement de leur poste de travail ?

Il est démontré en effet que la promotion de saines habitudes de vie sur le lieu de travail peut avoir des effets bénéfiques pour améliorer la santé globale des employés, et de façon indirecte la santé des familles, et qu'elle contribue également à une meilleure productivité de l'entreprise.

II- LES OBJECTIFS DE LA COALITION

Le programme ***Better Hearts, Better Cities***, est une initiative globale financé par la Fondation Novartis et exécuté par PATH, Intrahealth et le CRDH. Cette initiative également mené dans les villes de Sao Paulo au brésil et à Ulaanbaatar en Mongolie a pour but de soutenir le Ministère de la Santé et de l'Action Sociale dans sa lutte contre les MNT et en particulier l'HTA. PATH, est chargé de mobiliser les entreprises à rejoindre les efforts globaux du Sénégal en ce sens.

Ainsi, la coalition sera pour les entreprises, un espace d'échange et de partage d'expérience sur la faisabilité et la mise en œuvre de programmes de santé et bien-être dans les lieux de travail impliquant la lutte contre l'HTA.

PATH assure le pilotage et la coordination des activités de sensibilisation, de prévention et de plaidoyer de la coalition pour lutter contre l'HTA.

PATH en collaboration avec les entreprises membres et les partenaires est chargé d'animer le réseau, et de définir les règles d'actions et d'organisation en vue de sa pérennisation.

PATH développe du matériel de prévention liés aux facteurs de risque de l'HTA et le partage avec les entreprises pour le déroulement de leurs programmes de santé et bien-être au travail.

CHARTE D'ENGAGEMENT POUR LA LUTTE CONTRE L'HYPERTENSION ARTÉRIELLE ET LA PROMOTION DE SAINES HABITUDES DE VIE

Cette charte d'engagement offre l'opportunité aux entreprises publiques et privées ou aux établissements scolaires, sur une base volontaire, de s'inscrire dans une démarche citoyenne et de responsabilité sociétale en proposant à leurs salariés des actions et un environnement allant dans le sens des objectifs de santé publique du pays (lutter contre l'hypertension artérielle en promouvant les saines habitudes de vie).

En signant cette charte, l'entité s'engage à :

ARTICLE 1 : Créer un comité de santé (CS) représentatif de l'ensemble des employés de l'entreprise, de l'organisation ou de l'établissement scolaire, chargé de mettre en œuvre des actions adaptées afin de promouvoir auprès des salariés de saines habitudes (alimentaires, pratique régulière d'activité physique). Le CS est également référent du projet de lutte contre l'hypertension artérielle et la promotion de saines habitudes de vie auprès de la direction, et à ce titre, l'informe chaque année des actions mises en place et de celle prévues pour l'année suivante.

ARTICLE 2 : Créer des conditions favorables à l'adoption de comportements alimentaires sains sur le lieu de travail ou d'apprentissage pour les établissements scolaires, (offre alimentaire, aménagement du lieu de restauration, affichage informatifs, incitatifs, autres…) tout en privilégiant la convivialité. Le cas échéant, informer/guider les salariés /élèves vers des choix alimentaires les plus favorables au maintien d'une bonne santé (informations, conseils, animations, ateliers découverte, autres…).

ARTICLE 3 : Encourager la pratique d'une activité physique régulière par l'organisation de séances d'activité physique au sein de l'entreprise ou de l'établissement scolaire, la mise en place de dispositifs incitatifs ou la participation à différents évènements sportifs organisés par des associations et fédérations locales.

ARTICLE 4 : Créer un environnement favorable à l'arrêt du tabac par l'application de la règlementation interdisant l'usage du tabac dans les lieux publics.

ARTICLE 5 : Mettre en place chaque année, un minimum de 2 actions dans un des domaines d'interventions liés aux facteurs de risque modifiables de l'hypertension artérielle :

- ✓ Célébration de la journée Mondiale de l'hypertension artérielle
- ✓ Alimentation équilibrée
- ✓ Activité physique
- ✓ Lutte contre le Tabac/ Stress
- ✓ Information-éducation-communication pour de saines habitudes de vie

ARTICLE 6 : Collaborer avec la médecine du travail, pour le suivi et la prise en charge des salariés hypertendus selon les nouveau normes et protocoles du programme Better Hearts Better Cities. Mettre en place des visites de suivi régulières pour le contrôle de la pression artérielle entre le médecin du travail et le salarié.

ARTICLE 7 : Collaborer avec la médecine du travail et les services des ressources humaines dans le cadre d'une démarche d'évaluation des actions menées afin d'identifier leur impact sur le comportement et la santé des salariés et le retour sur investissement du programme de lutte contre l'hypertension artérielle et la promotion de saines habitudes de vie. Les entreprises s'engagent à partager de manière trimestrielle les résultats (agrégés) sur le nombre d'employés dépistés, suivi, sous traitement, et contrôlé.

ARTICLE 8 : Promouvoir et communiquer sur la démarche de la coalition de lutte contre l'hypertension artérielle et la promotion de saines habitudes de vie de façon explicite sur les documents informant du programme ou des actions menées au sein et en dehors de l'entreprise.

ARTICLE 9 : Dans le cadre de la démarche de Responsabilité Sociétale des Entreprises (RSE) soutenir les efforts du pays auprès de la population (Ministère de la santé et de l'action sociale) pour la lutte contre l'hypertension artérielle et la promotion de saines habitudes de vie. Cette participation à l'effort globale peut se faire de plusieurs manières :

- ✓ Apporter des ressources techniques et ou financières pour l'organisation d'activités ou projets liés à la lutte contre l'hypertension artérielle et l'adoption de saines habitudes de vie auprès de la population.
- ✓ Lors des évènements et manifestations mettant à l'honneur les démarches de lutte contre l'hypertension artérielle et la promotion de saines habitudes de vie au sein des entreprises, établissements scolaires ou des services du pays, participer en tant qu'acteur afin de communiquer et promouvoir la démarche adoptée en interne.

Par ailleurs,

PATH dans le cadre de la coalition et du programme Better Hearts, Better Cities mettra à disposition des entreprises, organisations et établissements scolaires signataires une plateforme d'échange en ligne afin de communiquer sur :

- ✓ Les actions mises en œuvre, transmises par les signataires pour permettre ainsi leur mutualisation
- ✓ Les évènements santé programmés sur l'année et auxquels les entreprises et établissements scolaires peuvent participer
- ✓ Les partenaires clés pour accompagner les entreprises dans leur démarche auprès des salariés ou élèves.

NOM DE L'ENTREPRISE/ORGANISATION/ETABLISSEMENT SCOLAIRE

DATE DE L'ENGAGEMENT

SIGNATAIRE (nom et titre)

SIGNATURE
