# Supplementary material for: Fostering cardiovascular health at work – case study from Senegal
Source: BMC Public Health. 2021 Jun 10;21:1108. doi: 10.1186/s12889-021-11109-9 (PMC8194249; doi:10.1186/s12889-021-11109-9)
Supplement: Supplementary file 2 — Additional file 1. [file 12889_2021_11109_MOESM1_ESM.zip › Notebook.pdf]

PATH

Ministère de la Santé et de l'Action Sociale

LES MALADIES NON-TRANSMISSIBLES : UN FARDEAU HUMAIN ET ÉCONOMIQUE TRÈS ÉLEVÉ

BLOC NOTES

BETTER **HEARTS**
BETTER **CITIES**

Toutes les informations dans le présent document proviennent du site officiel de l'OMS et de différentes sources certifiées.

PATH

HYPERTENSION ET DIABÈTE, TOUS CONCERNÉS !

INFORMATIONS PERSONNELLES

NOM..

PRÉNOM (S)...

ADRESSE..

TÉLÉPHONE..

PORTABLE...

ADRESSE BUREAU..

....

EMAIL..

FAX ....

A PRÉVENIR EN CAS D'URGENCE ....

Qu'appelle-t-on Maladies non transmissibles (MNT) ?

Les MNTs, sont une préoccupation de santé publique majeure, avec des complications sociales et économiques significatives. Quatre MNT (le diabète, les maladies cardiovasculaires et métaboliques, les cancers, et les maladies respiratoires chroniques) sont maintenant la cause principale de morbidité et de mortalité dans le monde. Selon l'Organisation Mondiale de la Santé (OMS), ces quatre groupes d'affection sont responsables de 80% des décès prématurés dus aux MNT.

Coût humain des MNT :

- Chaque année, plus de 40 millions de personnes meurent de MNT, soit 70% des décès dans le monde
- 15 millions de personnes âgées entre 30 et 70 ans meurent chaque année d'une MNT.
- 7 millions de ces décès prématurés surviennent dans les pays à revenus faibles et intermédiaires

Coût économique :

- Baisse de la productivité du fait de l'absentéisme au travail mais aussi parce que les gens tombent malades et meurent dans la force de l'âge
- Coût exorbitant de la prise en charge des maladies pour les individus, les familles, les entreprises et pour le système de santé.

NOTES

PATH

Le plan stratégique du Sénégal pour lutter contre les MNT

En 2017, les partenaires techniques et financiers, dont l'OMS et la Fondation Novartis, ont accompagné le Ministère de la Santé et de l'Action Sociale à travers sa division de lutte contre les Maladies Non Transmissibles (DLMNT) à élaborer un plan stratégique **intégré** de lutte contre les MNT.

Quelques résultats de l'enquête STEPS au Sénégal (2015)

• Hypertension artérielle : La prévalence est de 29,8%. Les femmes sont plus touchées. Elle est le risque cardio-vasculaire la plus fréquente.

• Diabète : La prévalence globale du diabète est de 3,2%. Les hommes sont plus touchés que les femmes. Le diabète est plus fréquent en zone urbaine qu'en zone rurale

• Le surpoids et l'obésité ont un taux de prévalence de 22%.

En 2017, une initiative de santé urbaine avec l'appui de la fondation Novartis, ''Better Hearts Better Cities'' est menée dans la ville de Dakar pour une meilleure prise en charge de l'hypertension artérielle.

NOTES

BETTER HEARTS BETTER CITIES

Better Hearts Better Cities est une initiative de santé urbaine de la Fondation Novartis qui a pour but d'améliorer la santé cardiovasculaire dans les pays à faibles revenus, et soutenue par le Ministère de la Santé et de l'Action Sociale dans le cadre de la Lutte contre les Maladies Non Transmissibles.

Il s'agit d'une approche globale et multisectorielle avec pour objectif l'amélioration de la santé cardio-vasculaire dans les communautés à faibles revenus.
Cette initiative est menée dans 3 villes, Ulaanbaatar en Mongolie, Dakar au Sénégal et Sao Paulo au Brésil.

BHBC SENEGAL est constitué d'un partenariat fort entre la Division de Lutte contre les Maladies Non-Transmissibles (DLMNT) du Ministère de la Santé et de l'Action Sociale et les partenaires de mise en œuvre PATH, Intrahealth et le Centre de recherche pour le développement humain (CRDH).

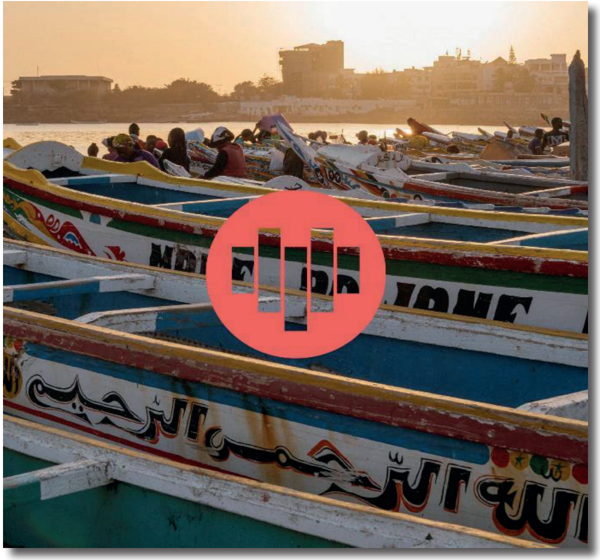

NOTES

PATH

Stratégie Multisectorielle à Dakar

- Renforcer la **qualité des soins de santé primaires** pour gérer l'hypertension
- Améliorer les capacités du patient à se prendre en charge et la **coordination des soins**
- Utiliser le patient **comme agent de changement** grâce à l'éducation
- Renforcer l'accès équitable aux médicaments
- Créer des **conditions de vie** et de travail plus saines

MNT et travail : focus sur le Workplace Program

Les MNT et leurs facteurs de risque ont un impact négatif sur le développement économique.

Les MNT diminuent la main-d'œuvre.

Les personnes touchées par les MNT sont le plus souvent dans leurs années les plus productives.

Pourtant, une main-d'œuvre en bonne santé est un moteur du développement économique.

Pour une entreprise, investir dans un programme de santé et de bien être est rentable.

NOTES

Qu'est ce que le diabète ?

• Le diabète est une maladie chronique qui ne se guérit pas, mais que l'on peut traiter et contrôler. Il provient d'un manque ou un défaut d'utilisation d'une **hormone** appelée **insuline.**

• L'insuline, produite par le **pancréas** permet au **glucose** (sucre) d'entrer dans les cellules du corps afin qu'il soit utilisé comme source d'énergie. Chez une personne non diabétique, l'insuline remplit bien son rôle et les cellules disposent de l'énergie dont elles ont besoin pour fonctionner.

• Lorsqu'il manque d'insuline ou que celle-ci ne parvient pas à accomplir sa fonction de façon efficace, comme c'est le cas chez une personne diabétique, le glucose ne peut pas servir de carburant aux cellules. Il s'accumule alors dans le sang et entraîne une augmentation du taux de sucre que nous allons appeler **hyperglycémie.**

• À long terme, un taux de sucre élevé dans le sang provoque certaines **complications,** notamment au niveau des yeux, des reins, des nerfs, du cœur et des vaisseaux sanguins.

• Il existe différents types de diabète tels que le **prédiabète**, le diabète **de type 1, de type 2, le diabète de grossesse et d'autres types plus rares.**

• Le diabète de type 1 (auparavant diabète juvénile) se définit par une production d'insuline insuffisante.

• La diabète de type 2 (diabète adulte) provient de l'utilisation inadéquate de l'insuline par l'organisme.

• Le diabète gestationnel concerne l'hyperglycémie qui est détectée pendant la grossesse.

NOTES

Qu'est ce que l'hypertension artérielle ?

L'hypertension artérielle, comme son nom l'indique, correspond à une forte pression du sang sur la paroi des artères, qui reste élevée quel que soit le contexte.

Symptômes et conséquences

L'hypertension, à un certain stade, peut se traduire par des maux de tête, des saignements de nez, des engourdissements, mais la plupart du temps il n'apparait aucun symptôme.

A long terme, l'hypertension artérielle est la cause de troubles cardiaques et vasculaires, d'insuffisance cardiaque ou rénale, ...

L'importance de la prévention

Faire connaître la pathologie et sensibiliser sur le dépistage, visent à la fois à éviter une hausse des personnes touchées et à faire en sorte que les personnes concernées puissent prendre connaissance de leurs statuts.

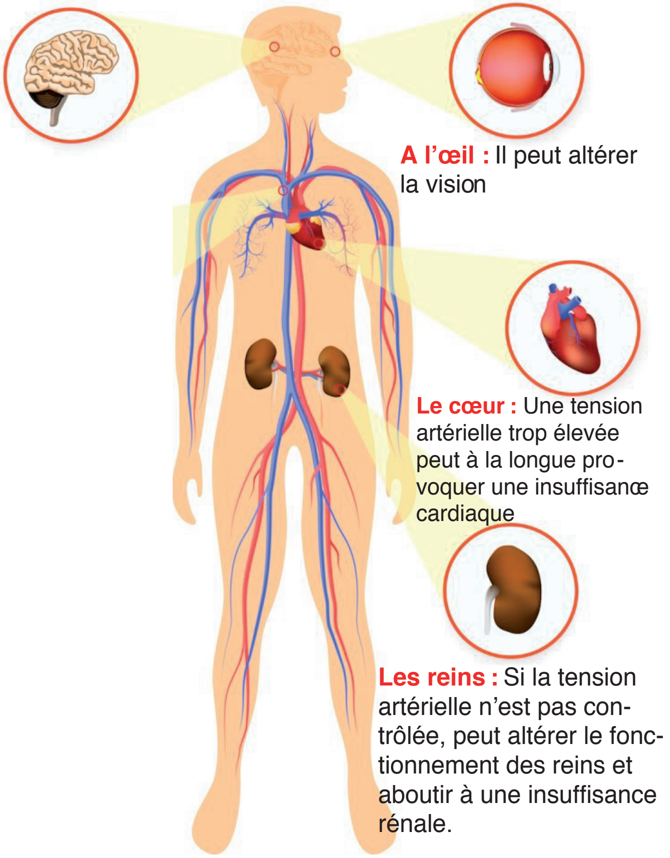

NOTES

GOOD to Know

Les différents niveaux de la tension artérielle

La **pression systolique** désigne la pression du sang lorsque le cœur se contracte et envoie le sang dans les artères. Elle assure un apport de sang partout à travers le corps.

La **pression diastolique** constitue la pression qui continue de s'exercer sur les artères entre chaque contraction. À ce moment, le cœur se détend et reprend son volume, ce qui permet aux cavités cardiaques de se **remplir** de sang.

Élevée

Systolique : Égale ou supérieure à 140 mmHg
Diastolique : Égale ou supérieure à 90 mmHg

Normal Haute

Systolique : entre 130 ou 139 mmHg
Diastolique : entre 85 et 89 mmHg

Normal

Systolique : entre 120 ou 129 mmHg
Diastolique : entre 80 et 84 mmHg

Optimale

Systolique : inférieure à 120 mmHg
Diastolique : inférieure à 80 mmHg

NOTES

PATH

HYPERTENSION ET DIABÈTE , TOUS CONCERNÉS !

Comment prévenir et contrôler l’hypertension artérielle et le diabète ?

Ces mesures vous permettront de prévenir et contrôler l’hypertension et le diabète :
Adopter une alimentation saine:

- Par la promotion d’une bonne nutrition notamment chez les jeunes
- Par la réduction de sa consommation de sel à moins de 5 g par jour (un peu moins d’une cuillère à café);
- En mangeant cinq portions de fruits et légumes par jour;
- Par la réduction de sa consommation de matière grasses

Limiter sa consommation journalière d’alcool à un verre standard maximum.
Pratiquer une activité physique :

- La pratique d’ une activité physique régulière
- Le maintien d’un poids normal : perdre 5 kg de surpoids peut réduire de 2 à 10 points la tension artérielle systolique.

Arrêter la consommation de tabac et l’exposition aux produits du tabac.
Suivre les recommandations médicales

Diagnostic et traitement du diabète et de l’hypertension :

On peut poser un diagnostic à l’aide d’un test sanguin qui est peu coûteux.
Les actions réalisables en cas de diabète sont les suivantes :

- Le contrôle de la glycémie, en particulier chez les personnes atteintes de diabète de type 1 qui ont besoin d’insuline; celles atteintes de diabète de type 2 peuvent être traitées par une médication par voie orale, mais peuvent également avoir besoin d’insuline;

- Le soin des pieds.

Le dépistage de l’hypertension passe, lui, par une mesure, régulière, de la tension artérielle, sachant qu’elle peut varier, en fonction des activités, ou d’une fois sur l’autre.

NOTES

PATH

HYPERTENSION ET DIABÈTE , TOUS CONCERNÉS !

Facteurs de risque de l’hypertension artérielle et du diabète

Les facteurs modifiables

Le Tabac

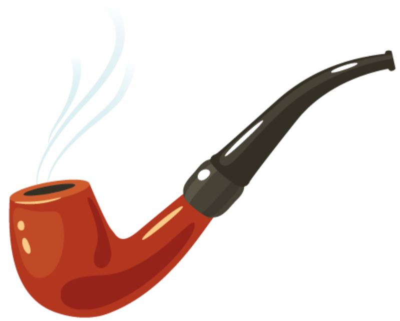

L’obésité

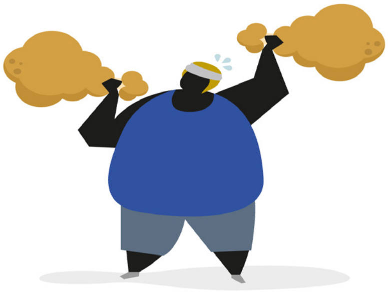

La sédentarité

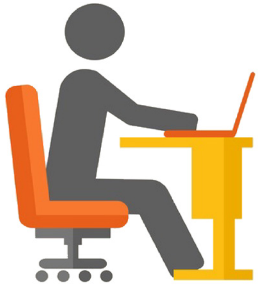

L’alcool

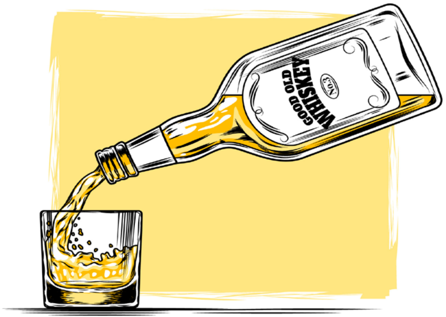

L’alimentation

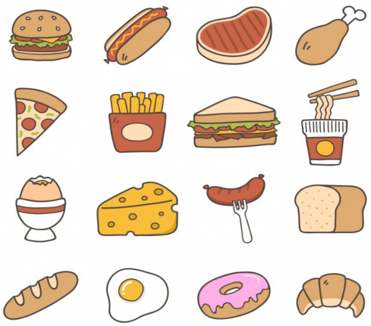

Stress

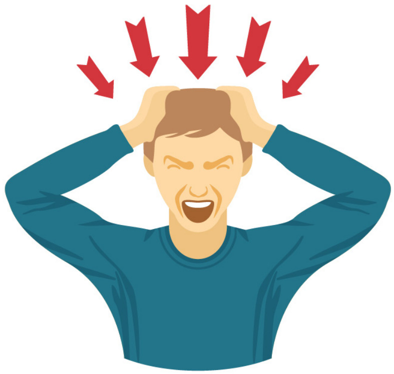

Les facteurs non modifiables

l’âge, le sexe, les antécédents personnels et ceux familiaux.

NOTES

BIEN- ÊTRE

1. Faire du sport

Faire du sport est aussi bénéfique pour la santé physique que pour celle mentale. L'activité du corps génère de l'endorphine, qui vous donne du plaisir et de l'euphorie. En plus de lutter contre la dépression, le permet de prévenir certaines maladies cardiovasculaires.

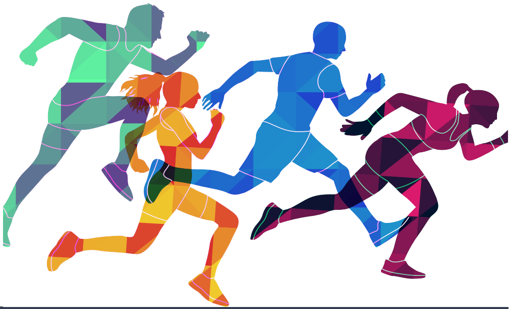

3. S'hydrater quotidiennement

L'hydratation est très importante dans pour un mode de vie sain.

Boire de l'eau facilite le transport des vitamines, des nutriments et des minéraux dans l'organisme. Sans même en être conscient, nous perdons plus de deux litres d'eau quotidiennement.
Il est donc recommandé de boire 1,5 litre d'eau par jour.

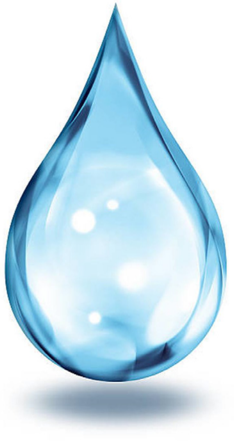

2. Manger sainement

Le fait d'avoir une alimentation saine et variée devrait être une des préoccupations majeures, puisque les aliments consommés procurent l'énergie nécessaire pour fonctionner.

Il est important de privilégier , la consommation de fruits et de légumes qui sera très bénéfique pour le corps.

Prendre la décision de manger sainement, c'est savoir respecter son corps.

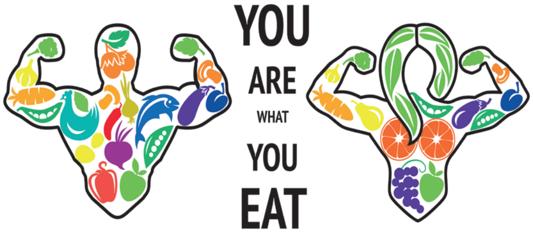

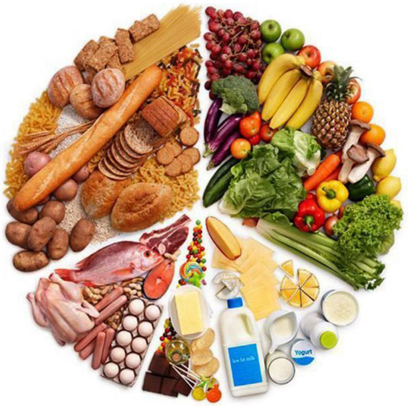

NOTES

PATH

HYPERTENSION ET DIABÈTE , TOUS CONCERNÉS !

BETTER HEARTS
BETTER CITIES

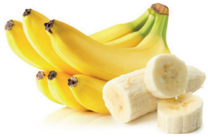

La banane est une excellente source de vitamines et contribue au maintien d'une bonne santé gastro-intestinale.

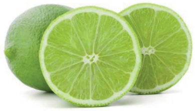

Le citron est un agrume peu calorifique et qui facilite la digestion.

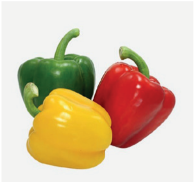

Riche en vitamines, le poivron stimule le transit intestinal et limite les risques de certains cancers.

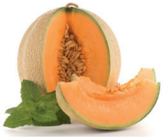

Le melon est une plante potagère, riche en eau et approprié pour la perte de poids.

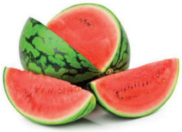

La pastèque aide à protéger les cellules des dommages liés au stress et diminue ainsi les risques de maladies cardio-vasculaires.

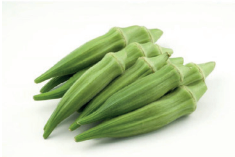

Le gombo est un aliment sain qui renforce votre immunité contre le diabète.

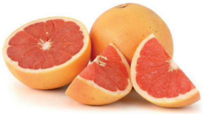

La pamplemousse possède de grandes vertus et permet de renforcer le système immunitaire.

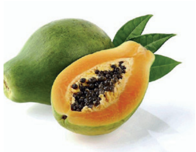

La papaye aide à la digestion et rééquilibre la flore intestinale. Ses fibres permettent de lutter contre les troubles du transit.

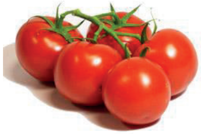

La consommation de tomates aurait des effets bénéfiques sur la prévention des maladies cardio-vasculaires.

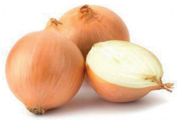

L'oignon est un aromate universel et agit sur différents facteurs de risques de maladies cardio-vasculaires .

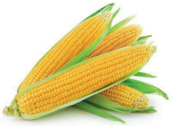

Le mais est une céréale atypique et constitue une source importante de fibres idéale pour les enfants et les femmes enceintes

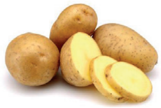

D'un apport nutritionnel important, la pomme de terre grâce à son amidon permet de prévenir certaines maladies inflammatoires.

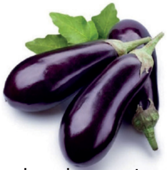

L'aubergine est composé de fibres douces qui participent au bon fonctionnement des intestins.

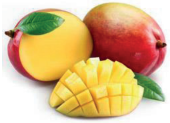

La mangue est le fruit qu'il vous faut pour lutter contre la mauvaise digestion et est un excellent laxatif.

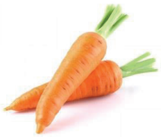

La carotte est un allié incontournable pour une bonne alimentation et est particulièrement recommandée pour les enfants.

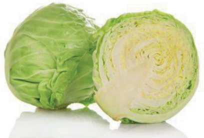

Le chou est un légume antianémique et régule également le métabolisme du sucre et des graisses.

PATH

HYPERTENSION ET DIABÈTE , TOUS CONCERNÉS !

BETTER **HEARTS**
BETTER **CITIES**

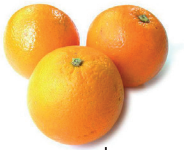

L'orange est une excellente source de vitamine C. Manger des oranges va alors permettre de stimuler le système immunitaire et lutter contre la fatigue comme les coups de froid hivernaux.

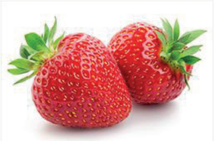

La fraise est peu calorique , riche en fibres et peut se consommer sans modération. Elle participe activement à la résistance des cellules et des tissus.

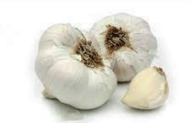

L'ail agit comme un antiseptique puissant du système digestif et de l'appareil respiratoire et aiderait également à la respiration.

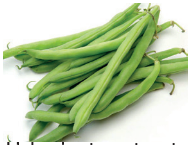

L' haricot vert est un légume de base de la cuisine,. Il ne contient pas beaucoup de calories et stimule le transit intestinal.

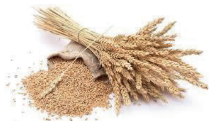

Pour une meilleure nutrition, les céréales doivent être complètes. Elles diminuent les graisses viscérales qui sont associées à l'hypertension et au diabète.

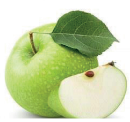

Qu'elle soit rouge ou verte, la pomme est l'un des fruits les plus consommés dans le monde. Elle permet de lutter contre l'apparition de certaines pathologies.

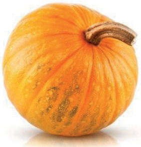

Le potiron est peu énergétique et peu calorique. Il est excellent pour la régulation de la pression artérielle , ce qui en fait un indispensable pour lutter contre l'hypertension.

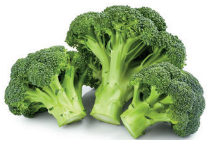

Le **brocoli** est un légume de la famille des **crufières.** Il renferme de **vitamine C. Les composés bioactifs** qu'il contient auraient des effets **anticancer.**

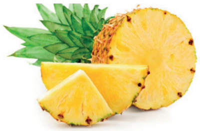

L 'ananas est constitué de vitamines et minéraux qui favorisent la circulation sanguine et a un effet protecteur contre certaines maladies dites dégénératives.

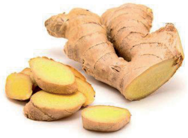

Le gingembre est une épice très appréciée dans le monde et est surtout conseillé dans le cas de manque d'énergie ou pour réguler la tension artérielle.

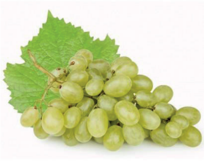

Le raisin est le fruit de l'énergie par excellence car il contient de nombreux sucres facilement assimilables

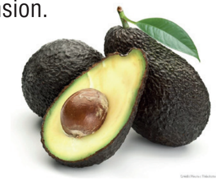

D'un apport important en fibres et en « bons gras » , l'avocat facilite le transit intestinal . Il est important de limiter sa consommation.

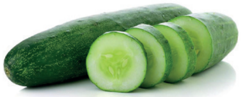

Il s'agit d'un légume rafraichissant avec une forte teneur en eau et qui peut être utilisé pour les pertes de poids.

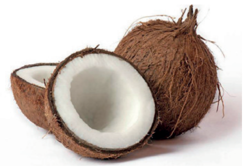

La noix de coco aide à stabiliser le sucre sanguin , facilite la digestion et permet de lutter contre l'inflammation. Elle procure également un sentiment de satiété.

NOTES

À propos de PATH

PATH est une organisation mondiale qui œuvre pour accélérer l'équité en matière de santé en réunissant des institutions, des entreprises, des entreprises sociales et solidaires et des investisseurs, pour résoudre les problèmes de santé mondiale les plus préoccupants. PATH dispose d'une expertise dans les domaines des sciences, de la santé, de l'économie, des nouvelles technologies et du plaidoyer ainsi que dans des dizaines d'autres spécialités lui permettant de développer et d'adapter diverses solutions. Parmi ces solutions figurent vaccins, médicaments, dispositifs médicaux, méthodes de diagnostic et approches innovantes pour renforcer les systèmes de santé à travers le monde.

PATH SENEGAL
Boite postale,
BP 15115, Dakar-Fann
Dakar, Sénégal

ADRESSE
Fann Résidence,
Rue Saint John Perse X F
Dakar, Sénégal
Tél.: +221 33 869 11 51

www.path.org
