# Supplementary material for: Fostering cardiovascular health at work – case study from Senegal
Source: BMC Public Health. 2021 Jun 10;21:1108. doi: 10.1186/s12889-021-11109-9 (PMC8194249; doi:10.1186/s12889-021-11109-9)

80/ TEE -SHIRTS

Devant

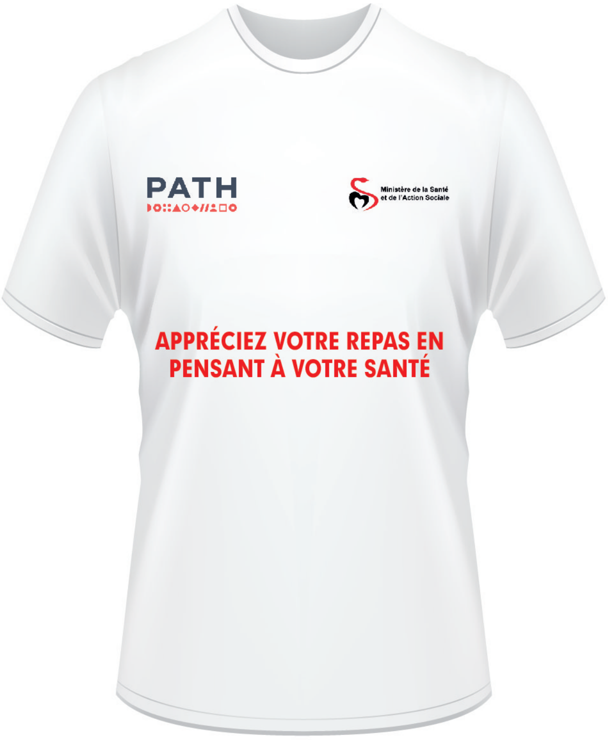

Dos

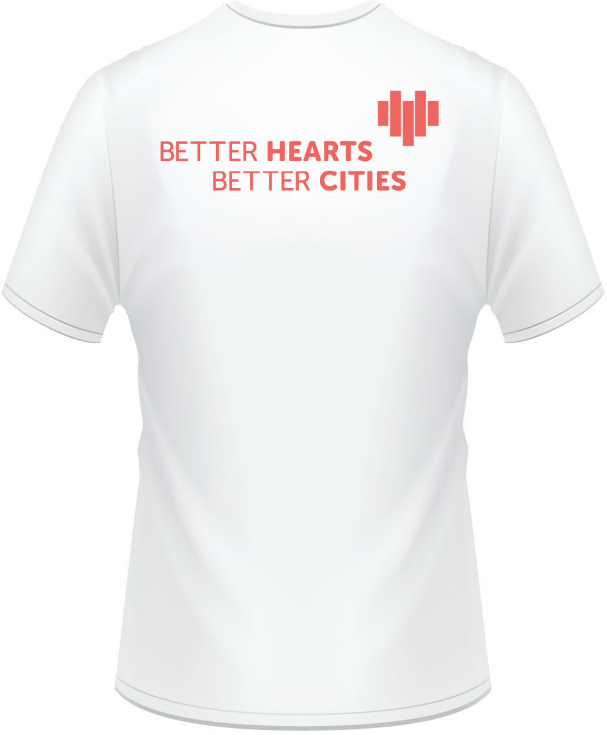

40/ POLOS

Devant

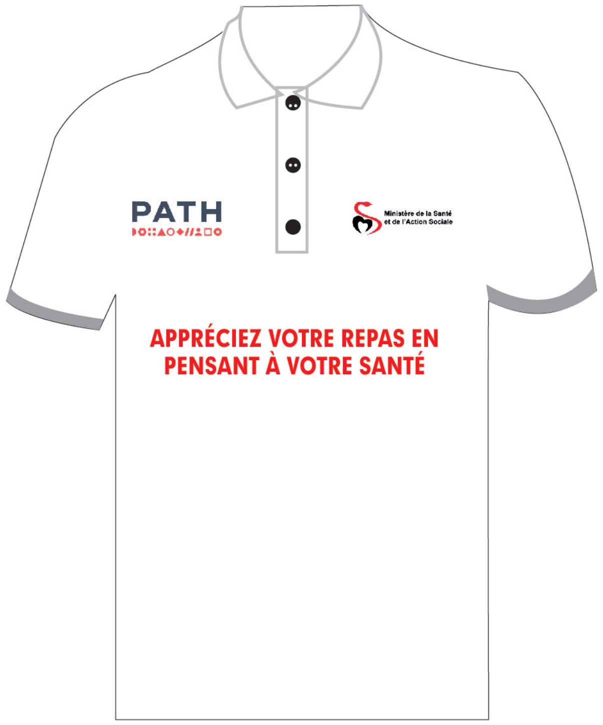

Dos

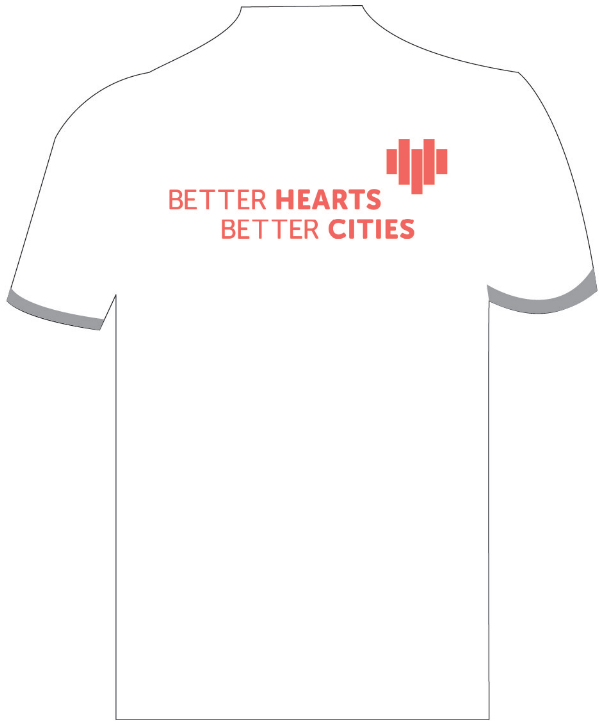

80/ TEE -SHIRTS

Devant

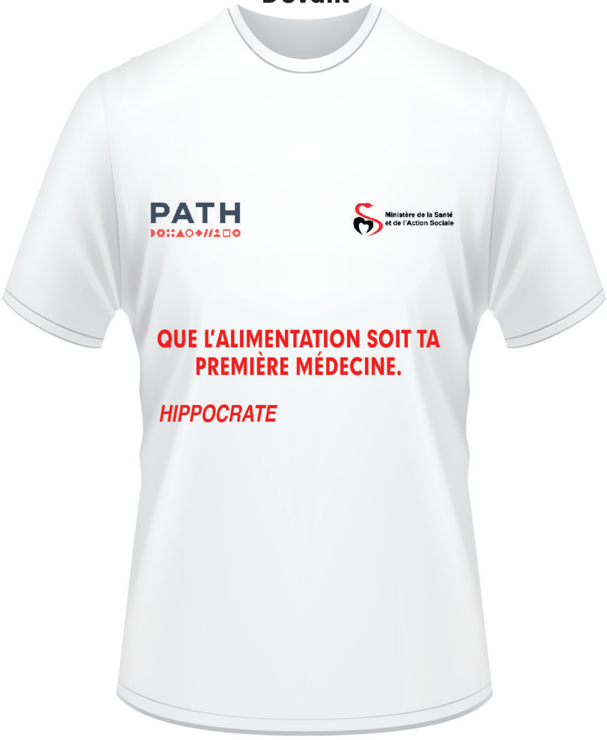

Dos

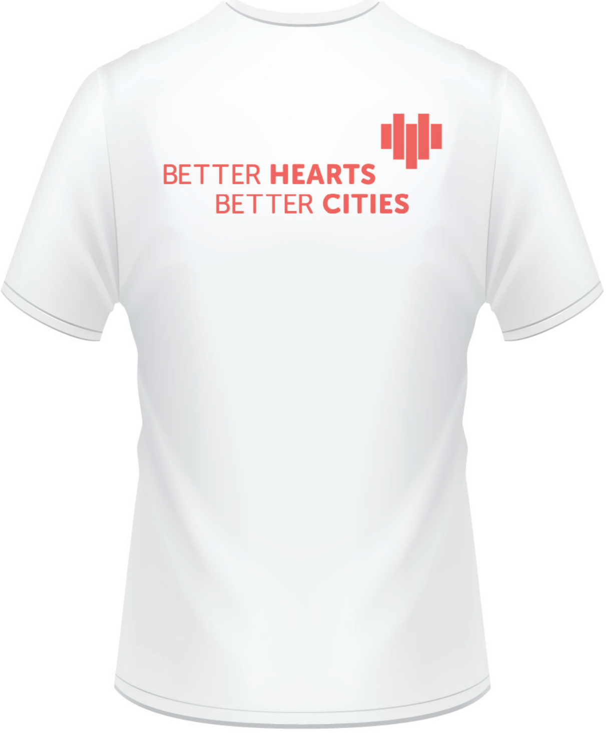

40/ POLOS

Devant

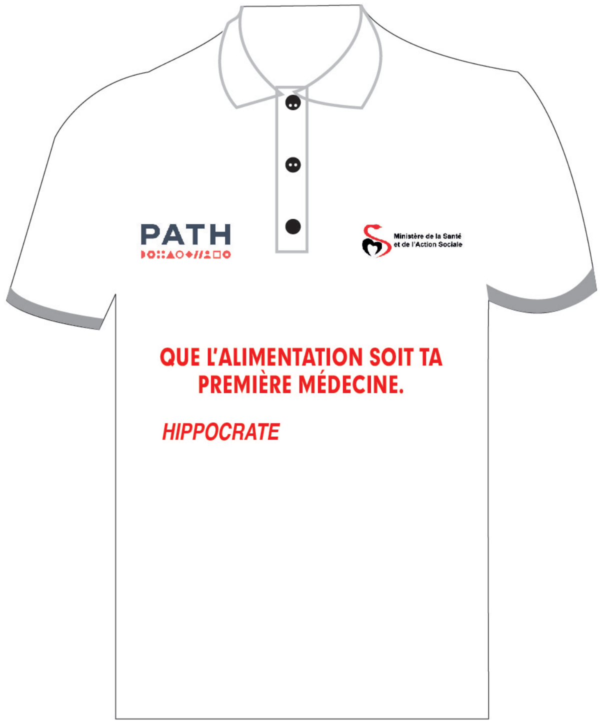

Dos

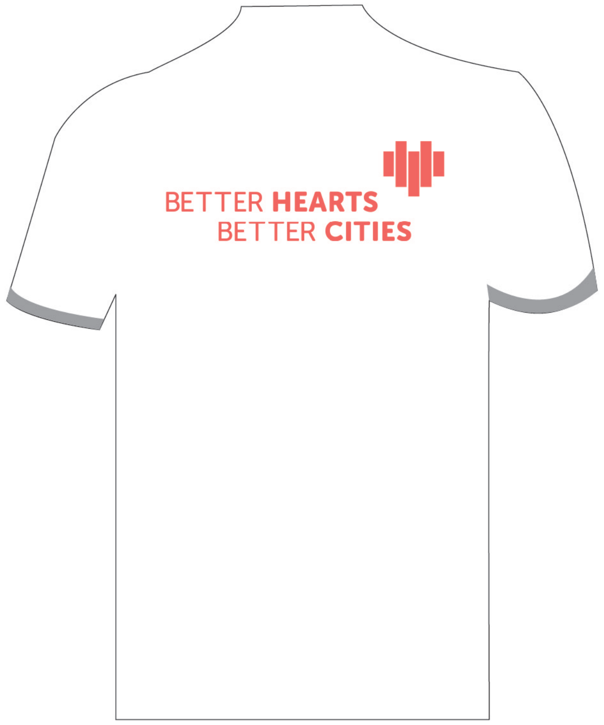

70/ TEE -SHIRTS

Devant

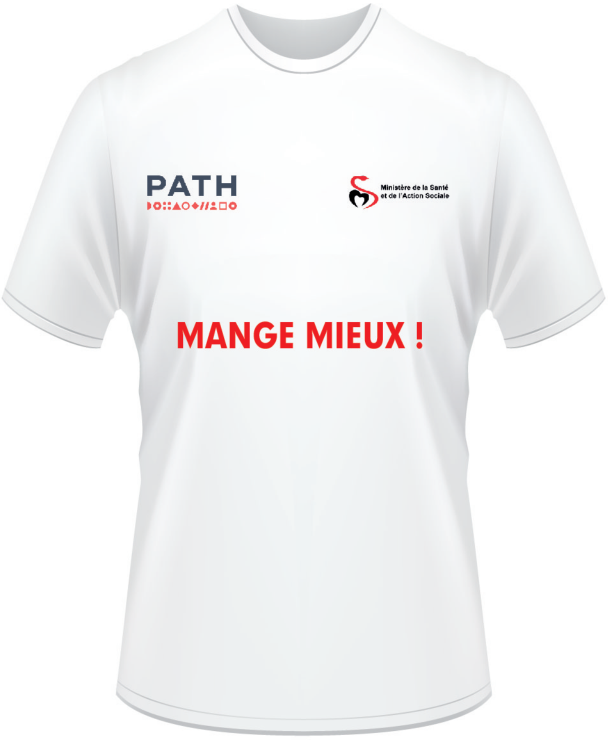

Dos

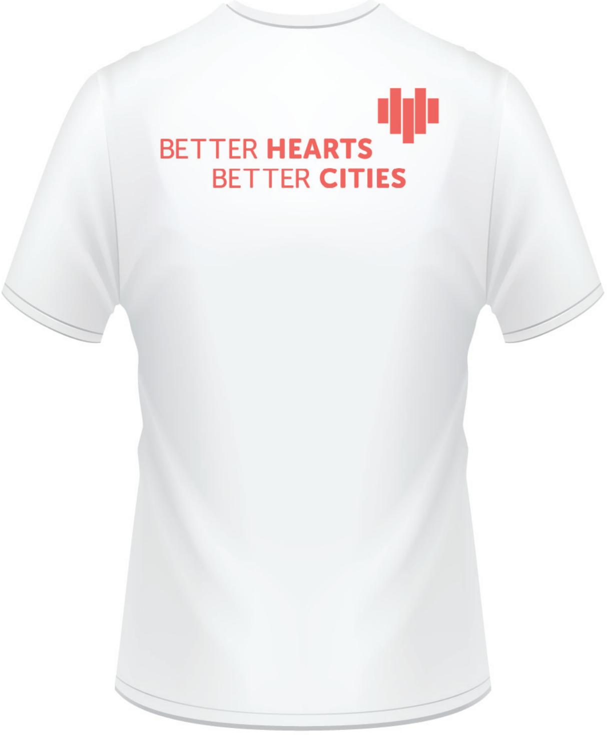

40/ POLOS

Devant

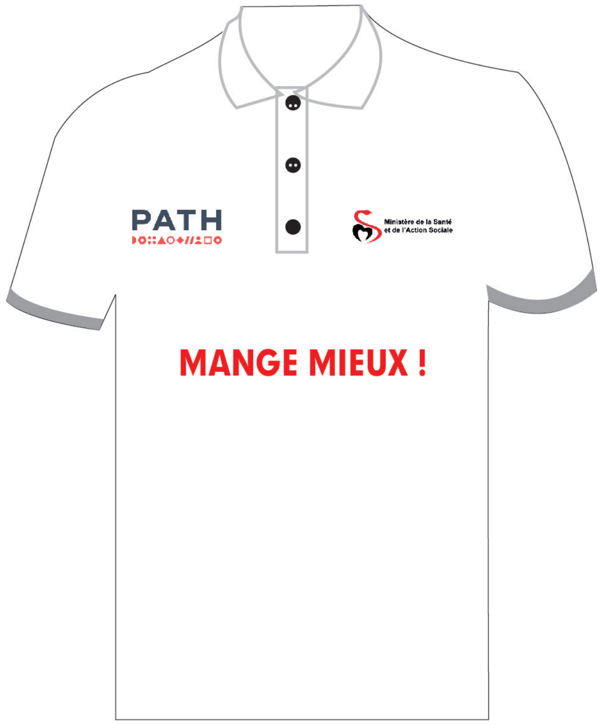

Dos

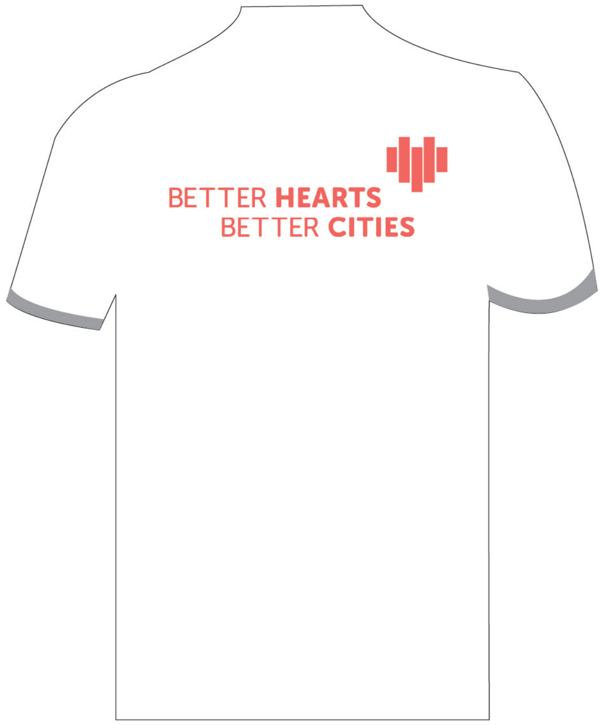

80/ TEE -SHIRTS

Devant

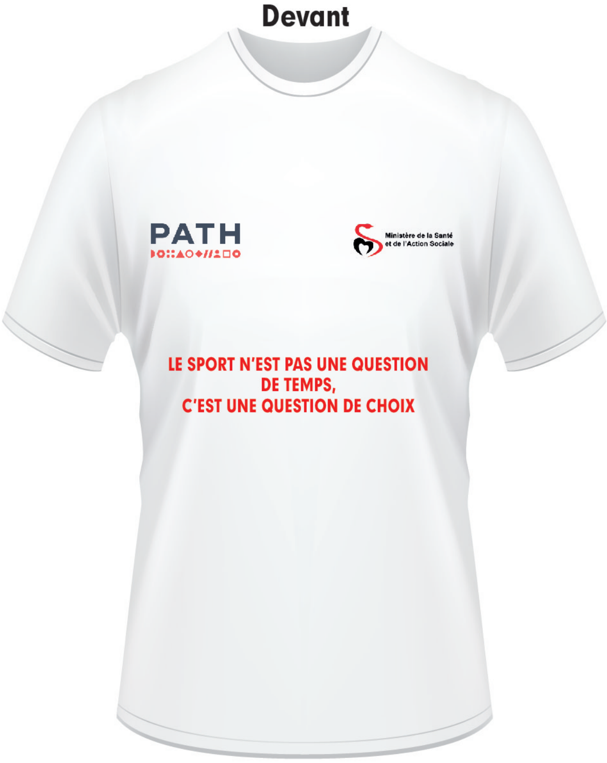

Dos

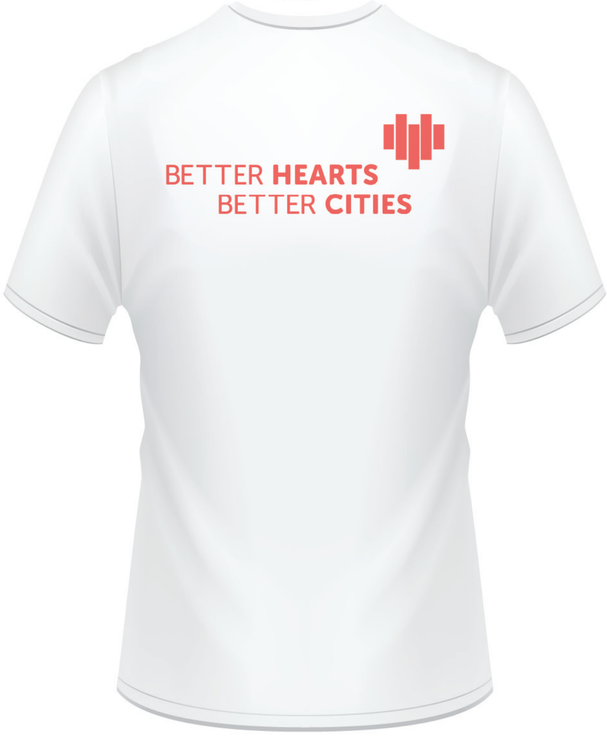

40/ POLOS

Devant

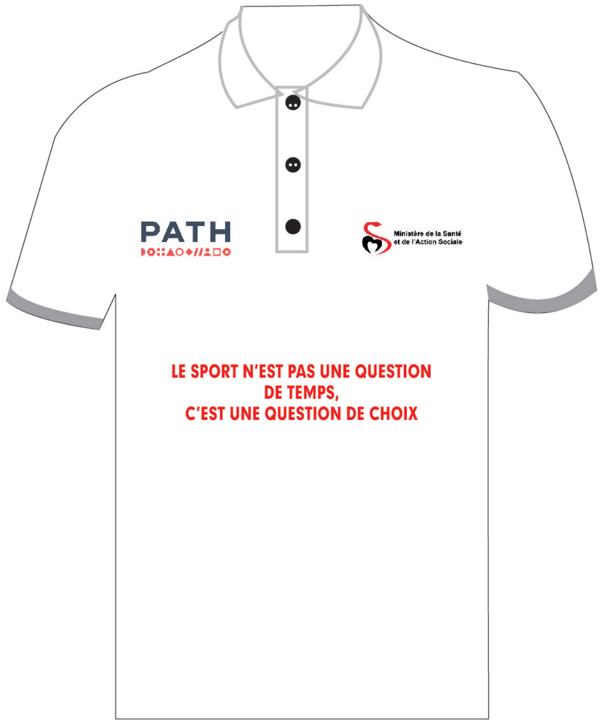

Dos

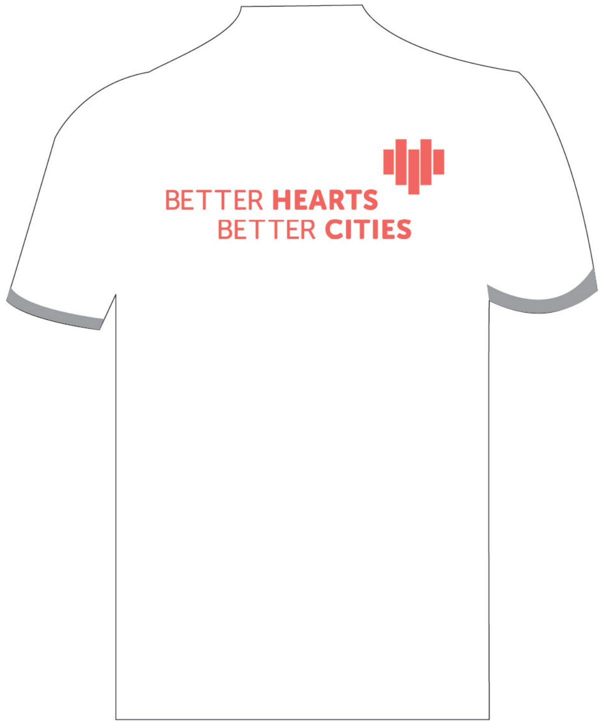

80/ TEE -SHIRTS

Devant

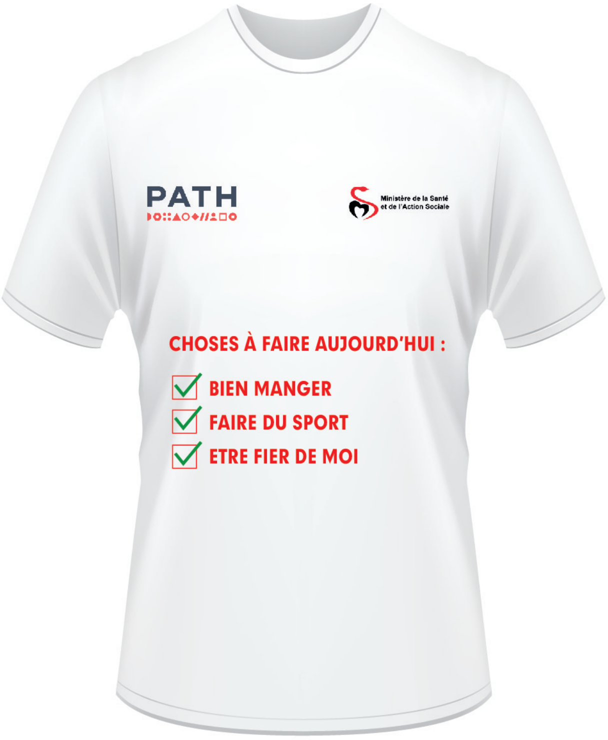

Dos

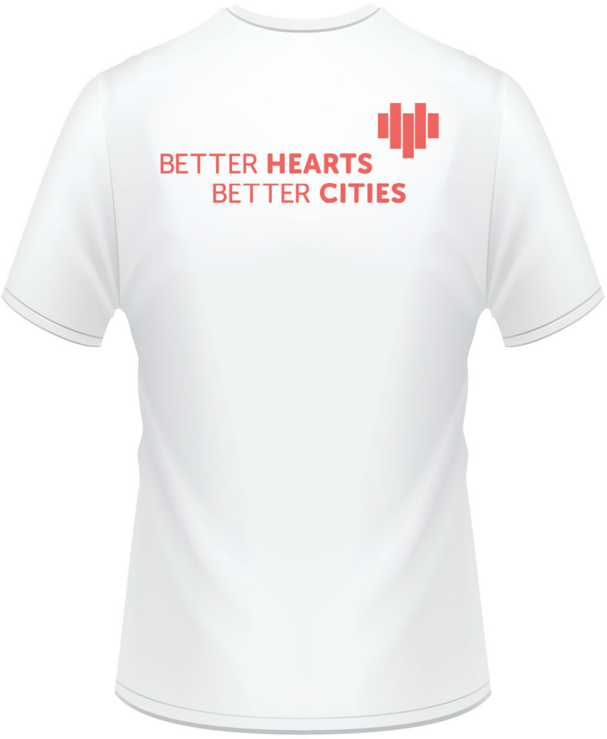

40/ POLOS

Devant

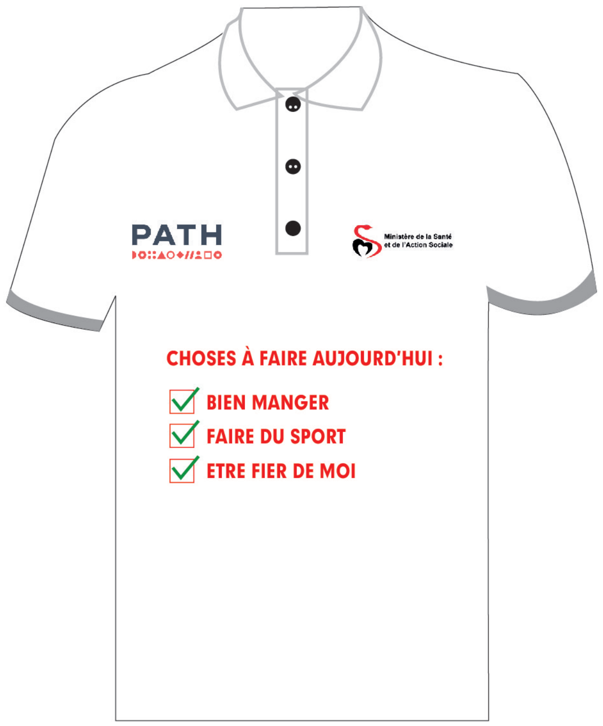

Dos

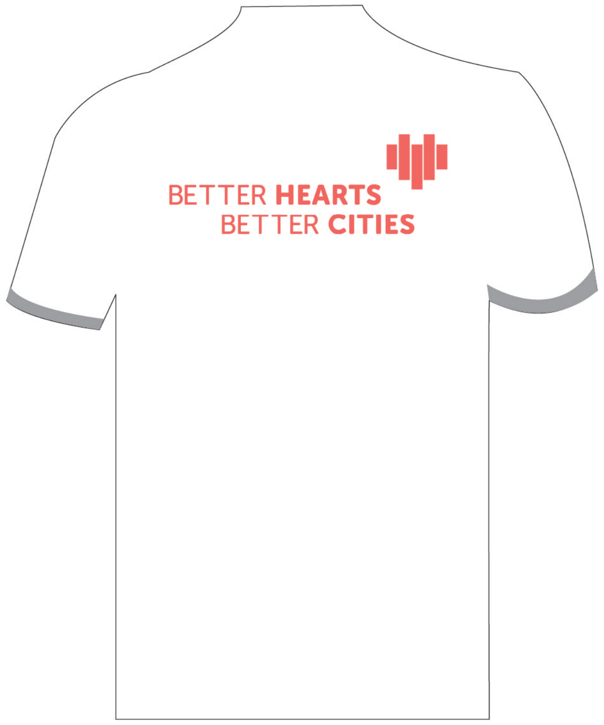

80/ TEE -SHIRTS

Devant

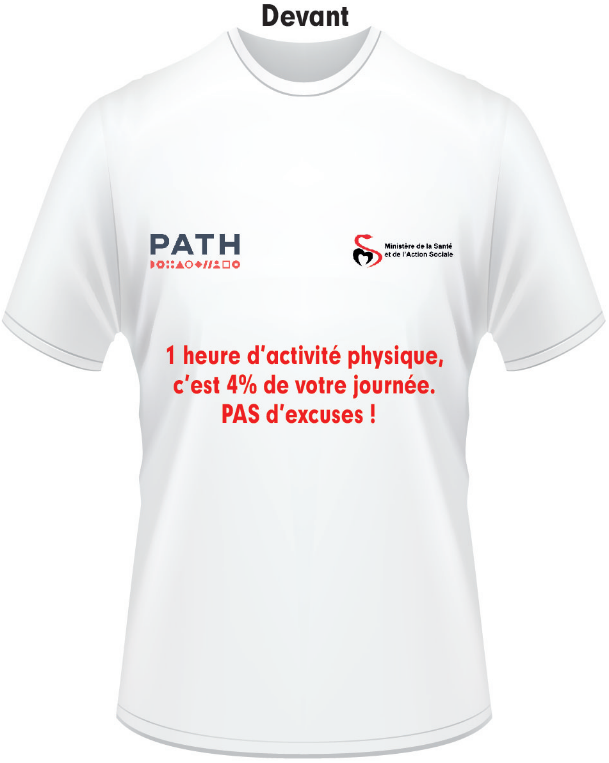

Dos

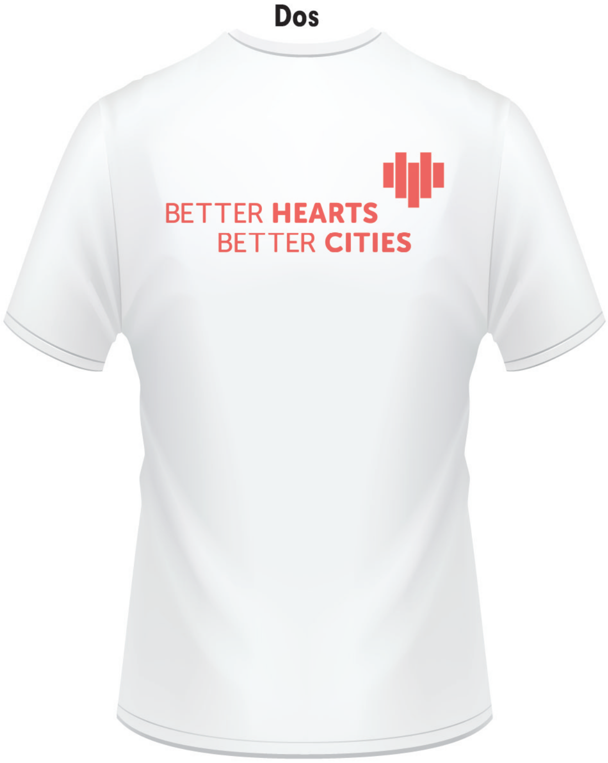

40/ POLOS

Devant

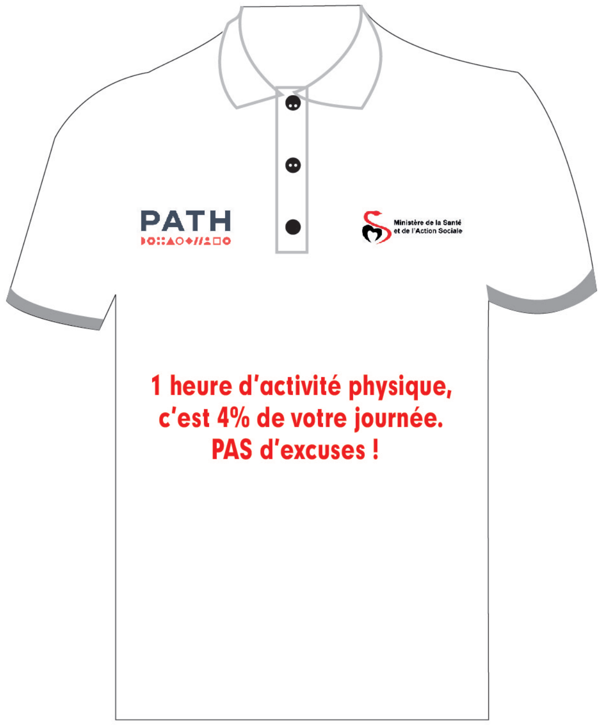

Dos

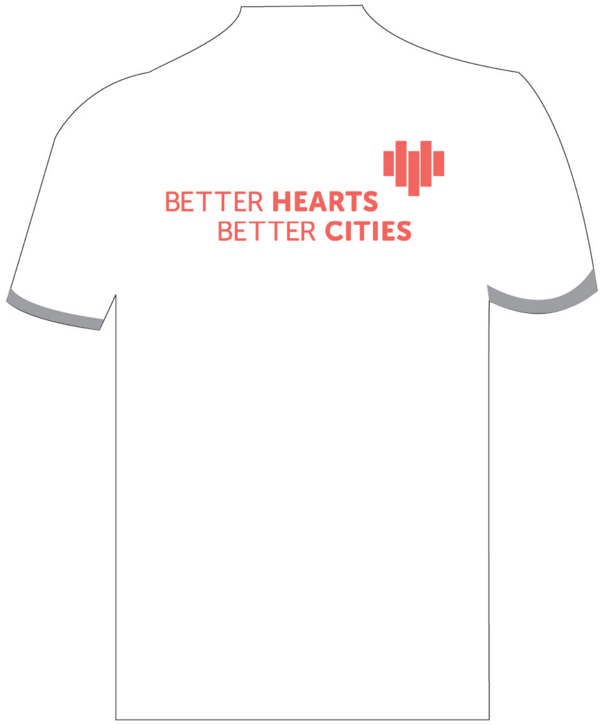

80/ TEE -SHIRTS

Devant

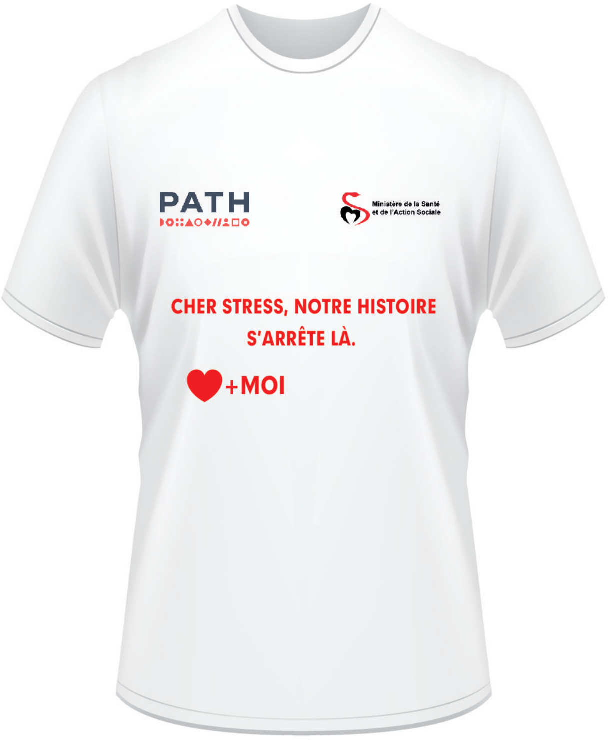

Dos

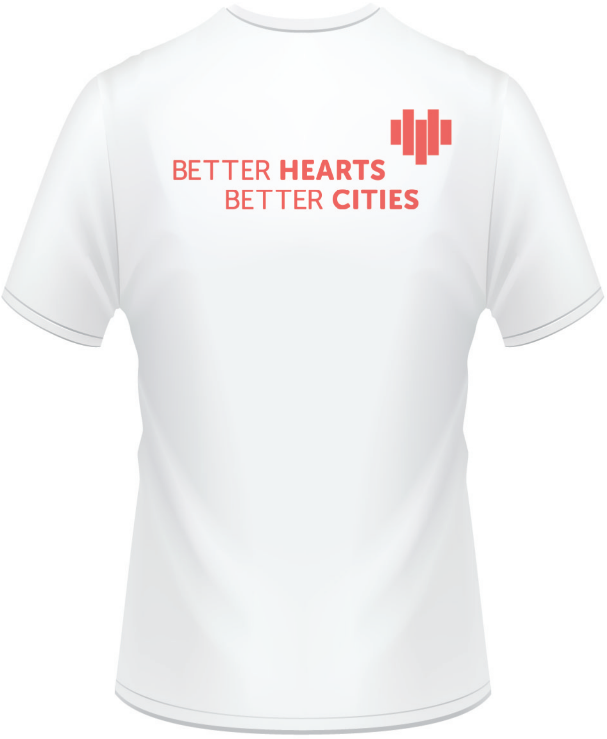

60/ POLOS

Devant

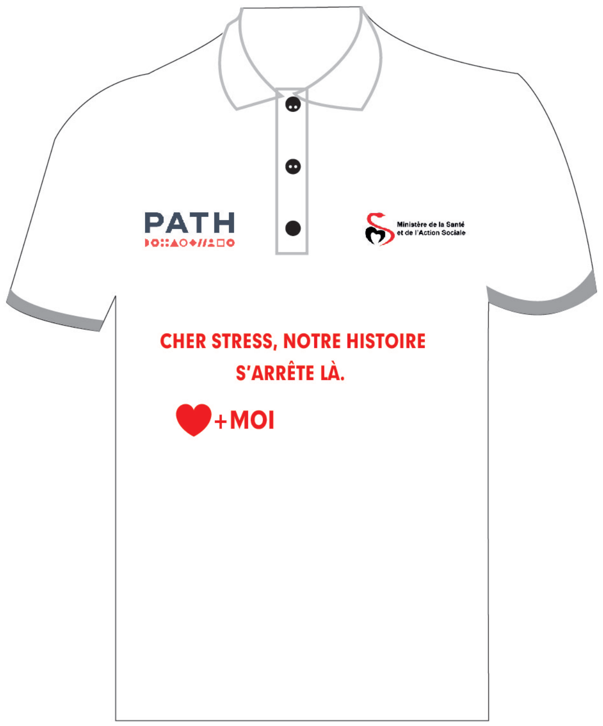

Dos

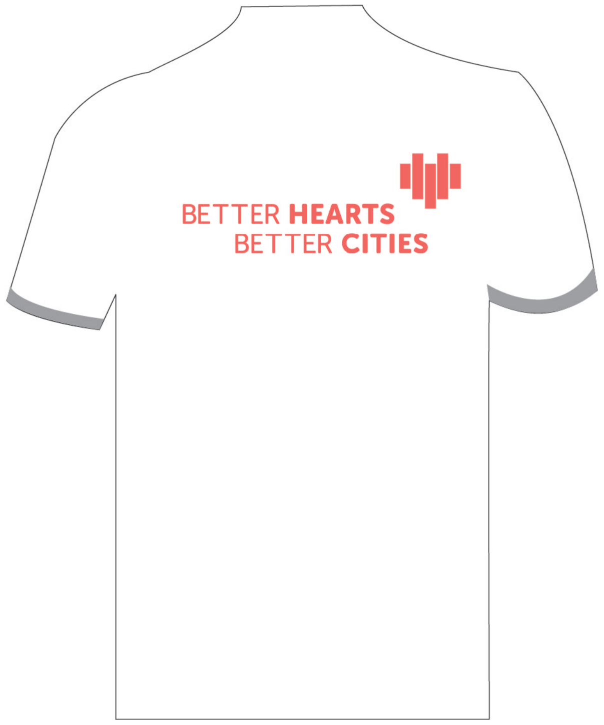

80/ TEE -SHIRTS

Devant

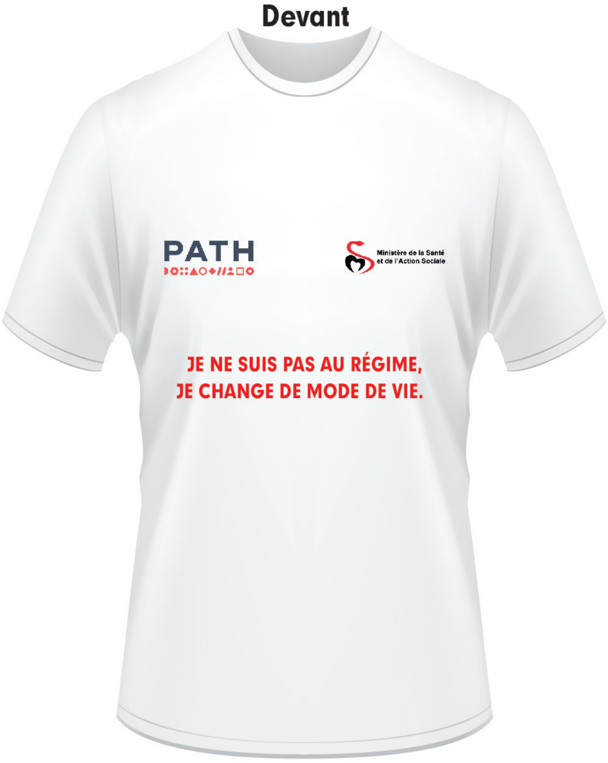

Dos

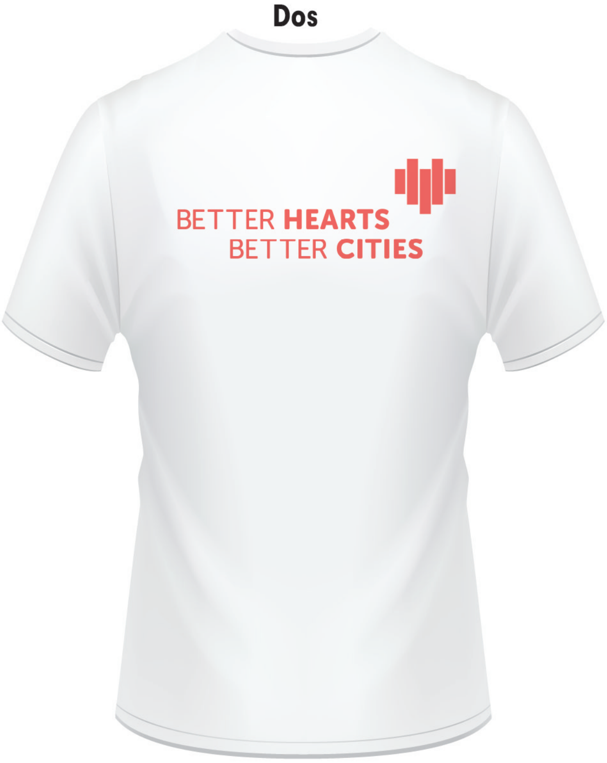

40/ POLOS

Devant

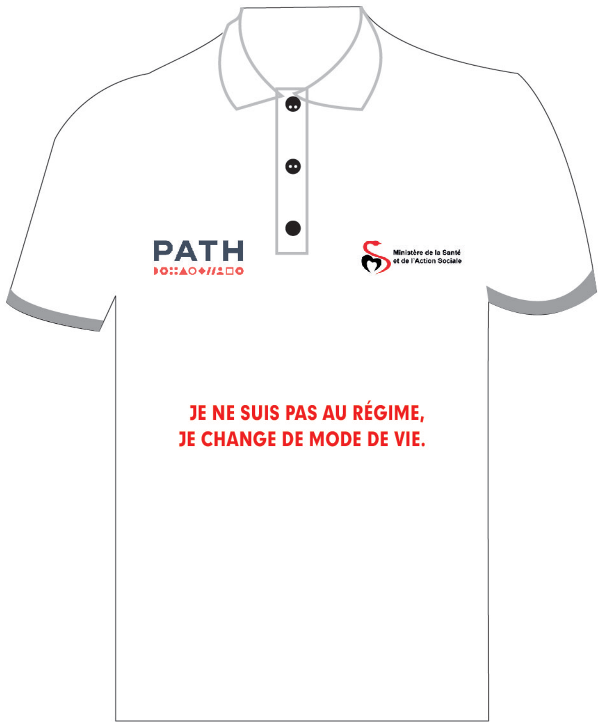

Dos

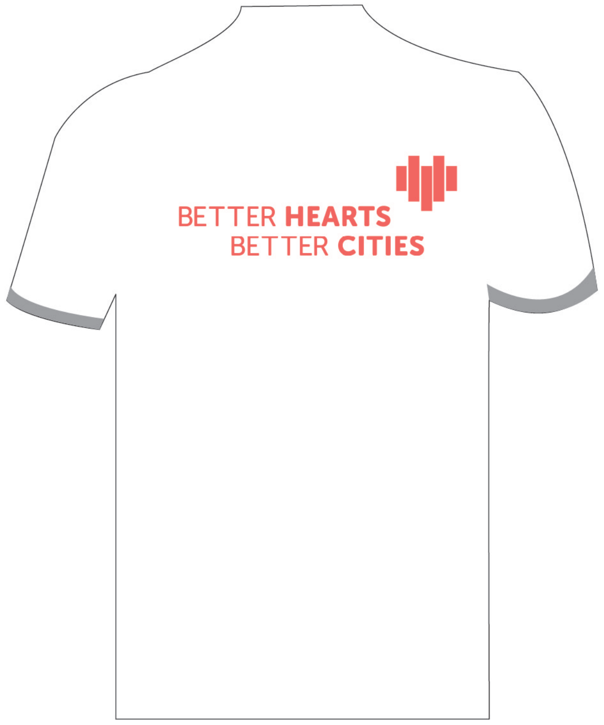

80/ TEE -SHIRTS

Devant

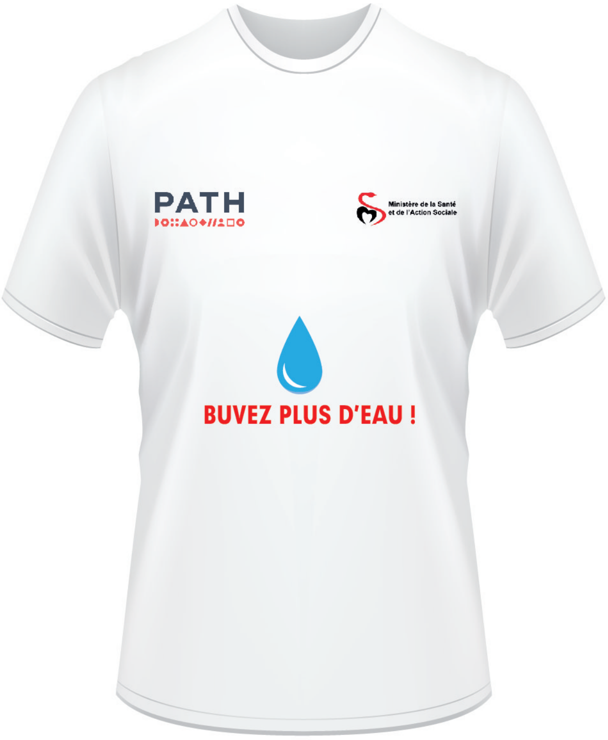

Dos

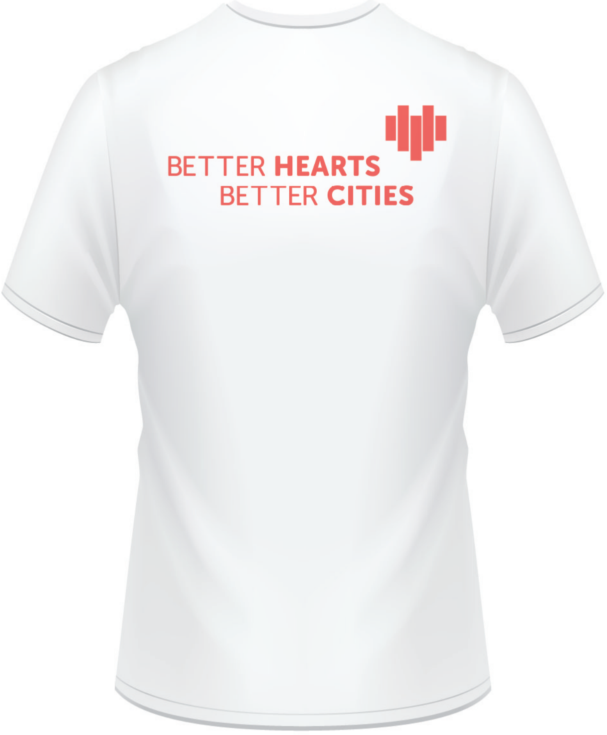

60/ POLOS

Devant

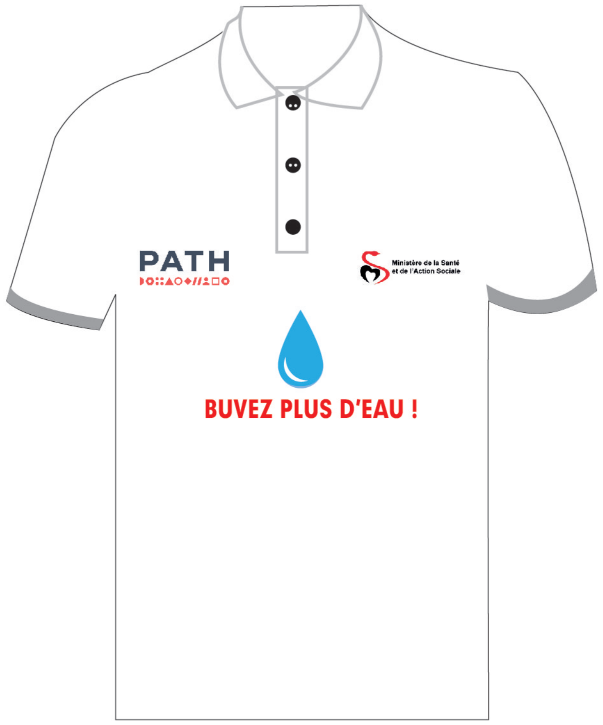

Dos

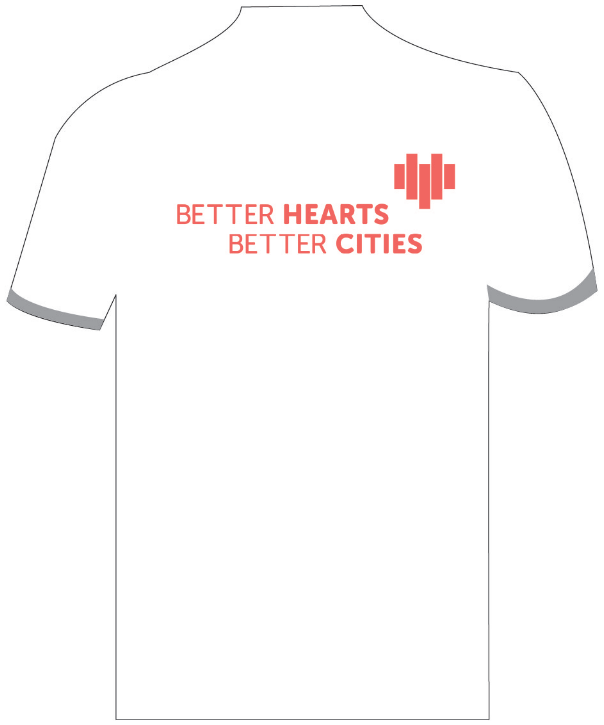

80/ TEE -SHIRTS

Devant

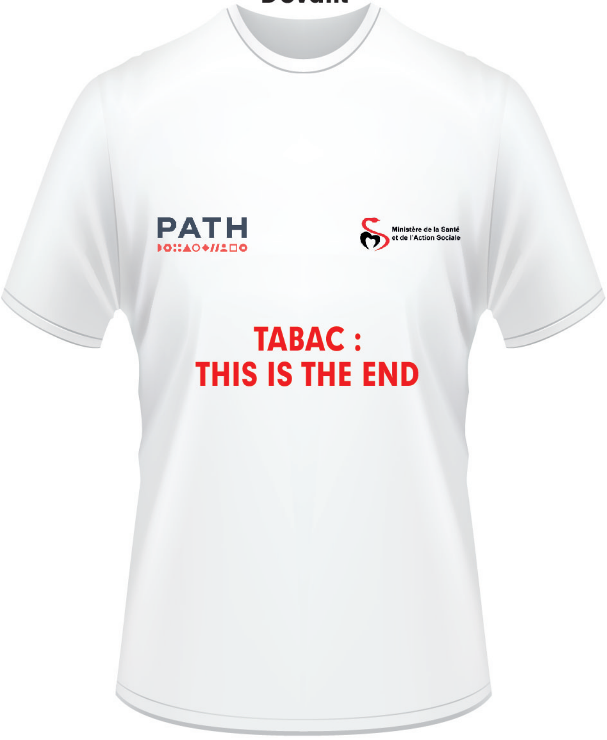

Dos

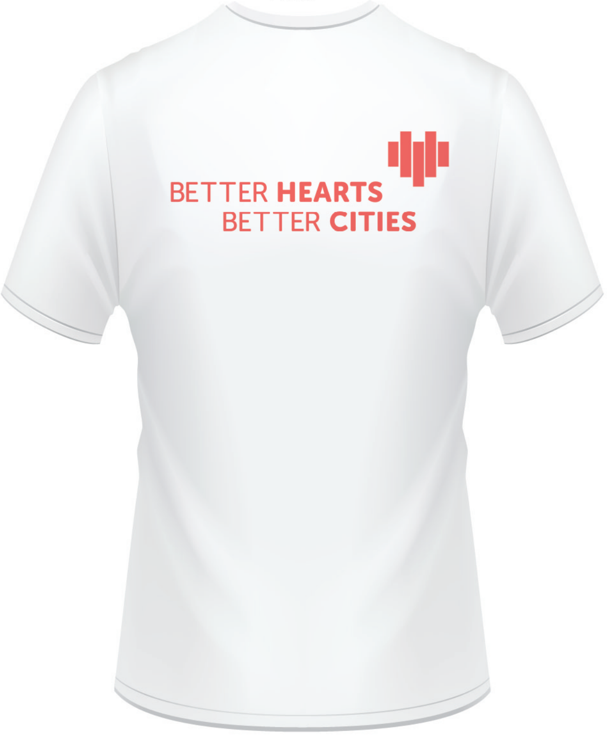

40/ POLOS

Devant

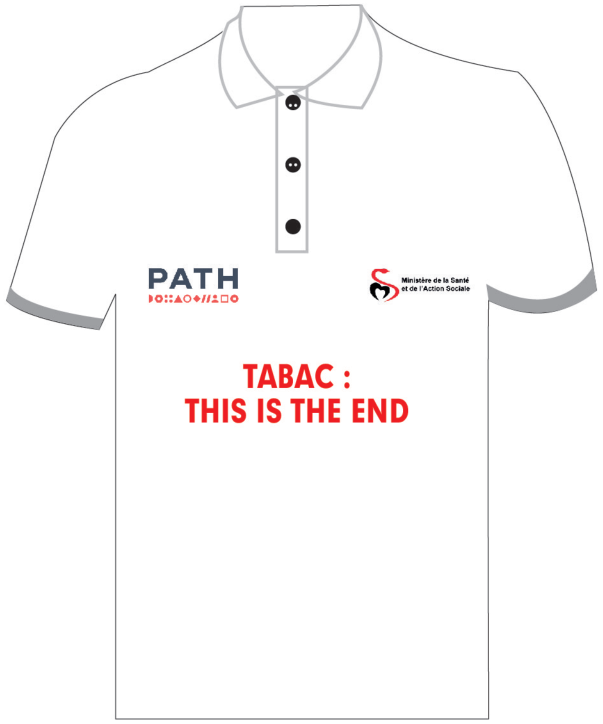

Dos

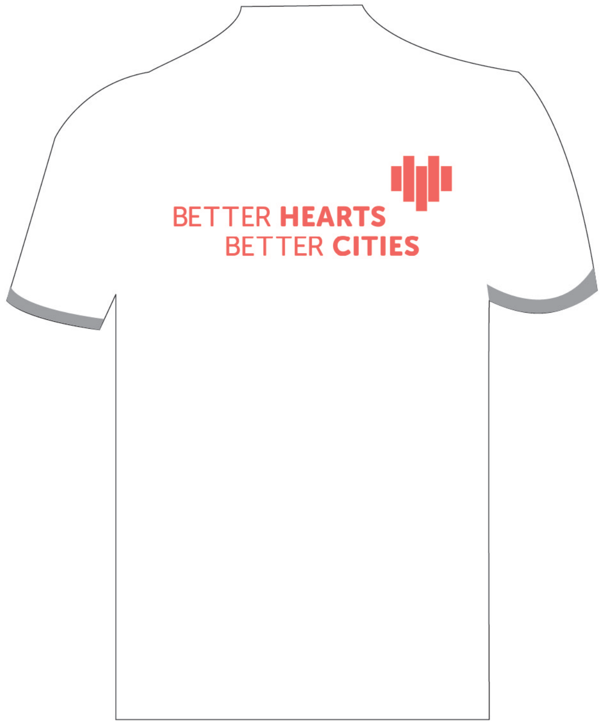

120/ TEE -SHIRTS

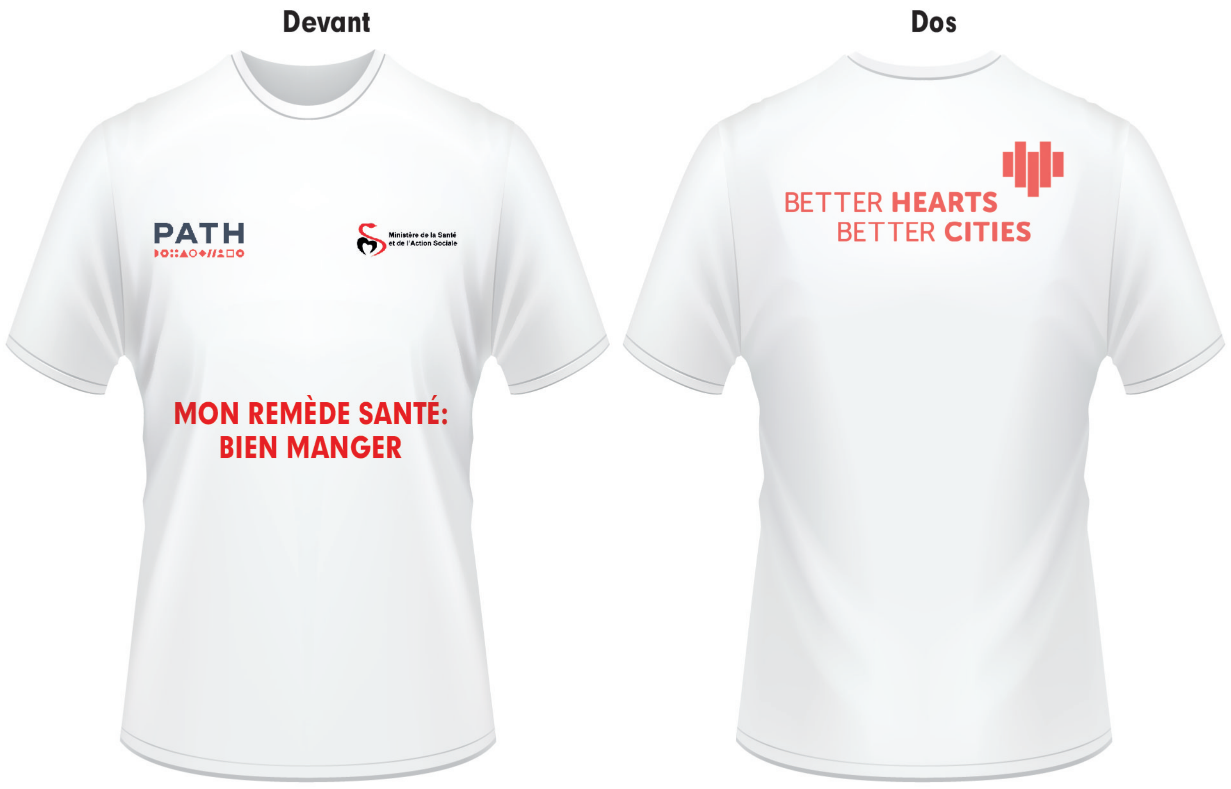

40/ POLOS

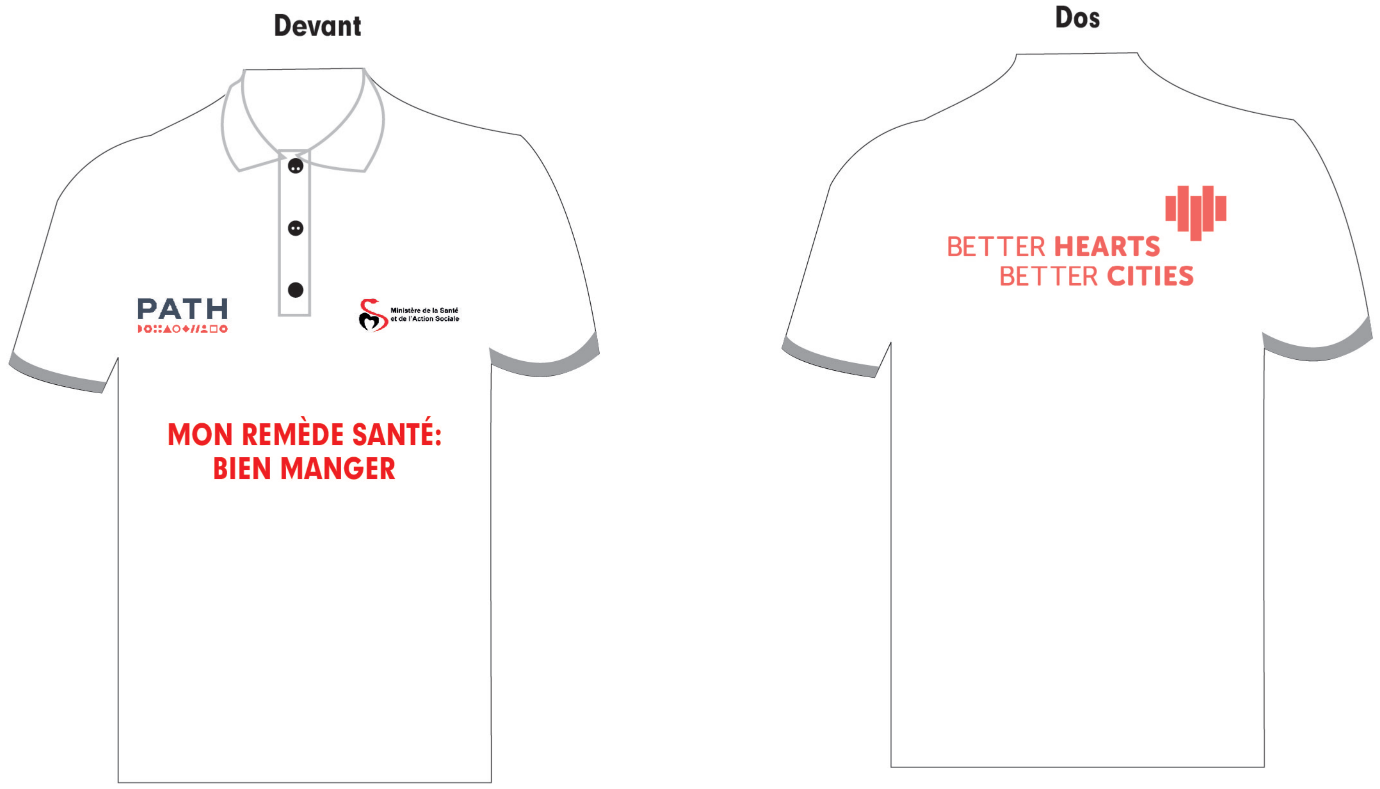

80/ TEE -SHIRTS

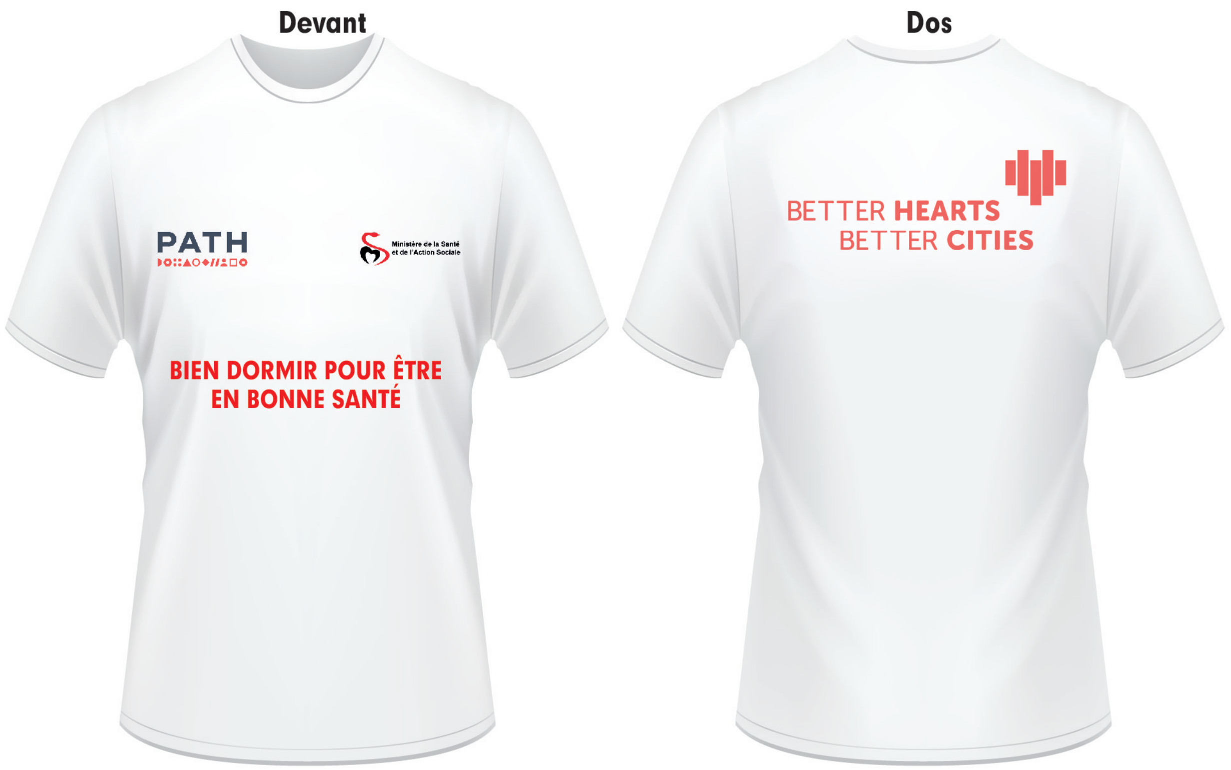

40/ POLOS

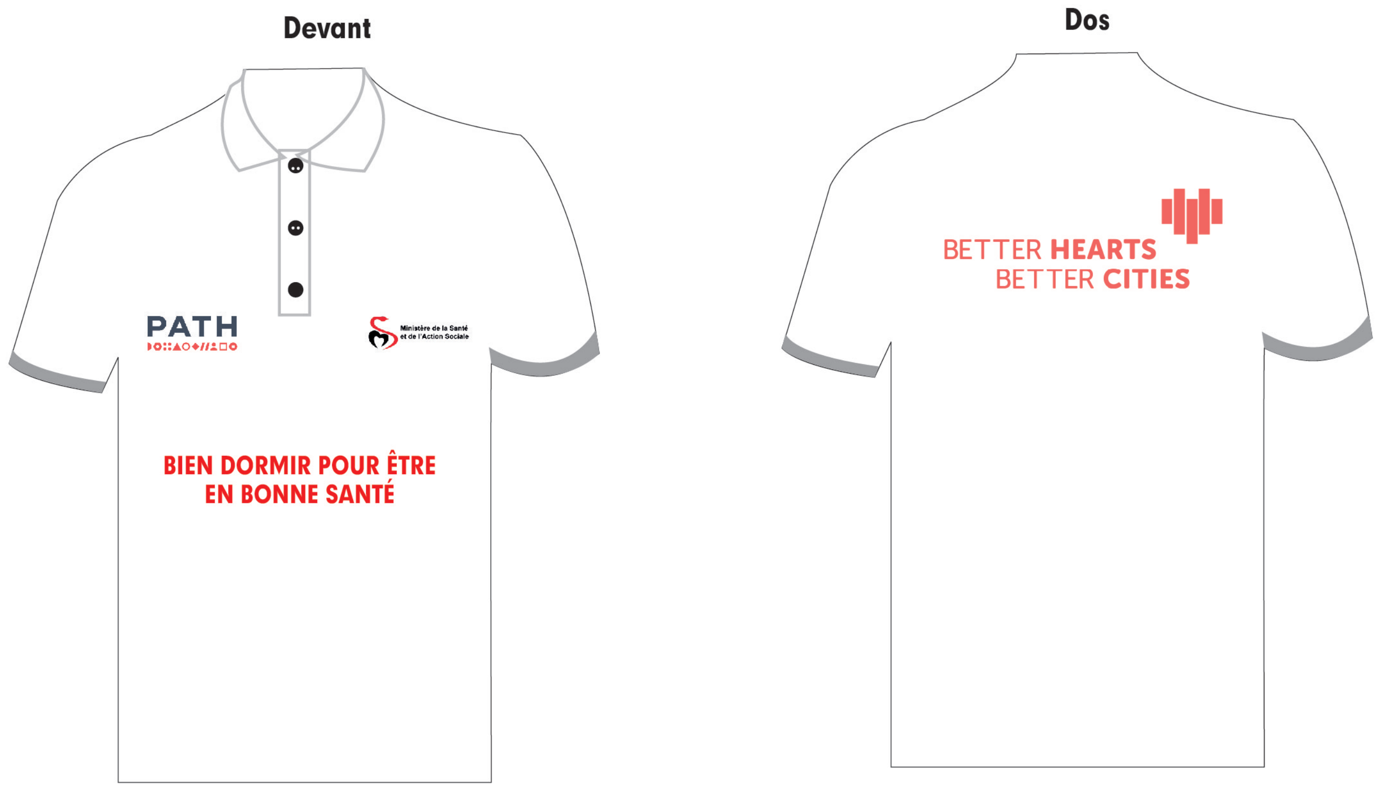

Supplement: Supplementary file 2 — Additional file 1. [file 12889_2021_11109_MOESM1_ESM.zip › T-Shirts.pdf]
