# Supplementary figures and images for: Fostering cardiovascular health at work – case study from Senegal
Source: BMC Public Health. 2021 Jun 10;21:1108. doi: 10.1186/s12889-021-11109-9 (PMC8194249; doi:10.1186/s12889-021-11109-9)

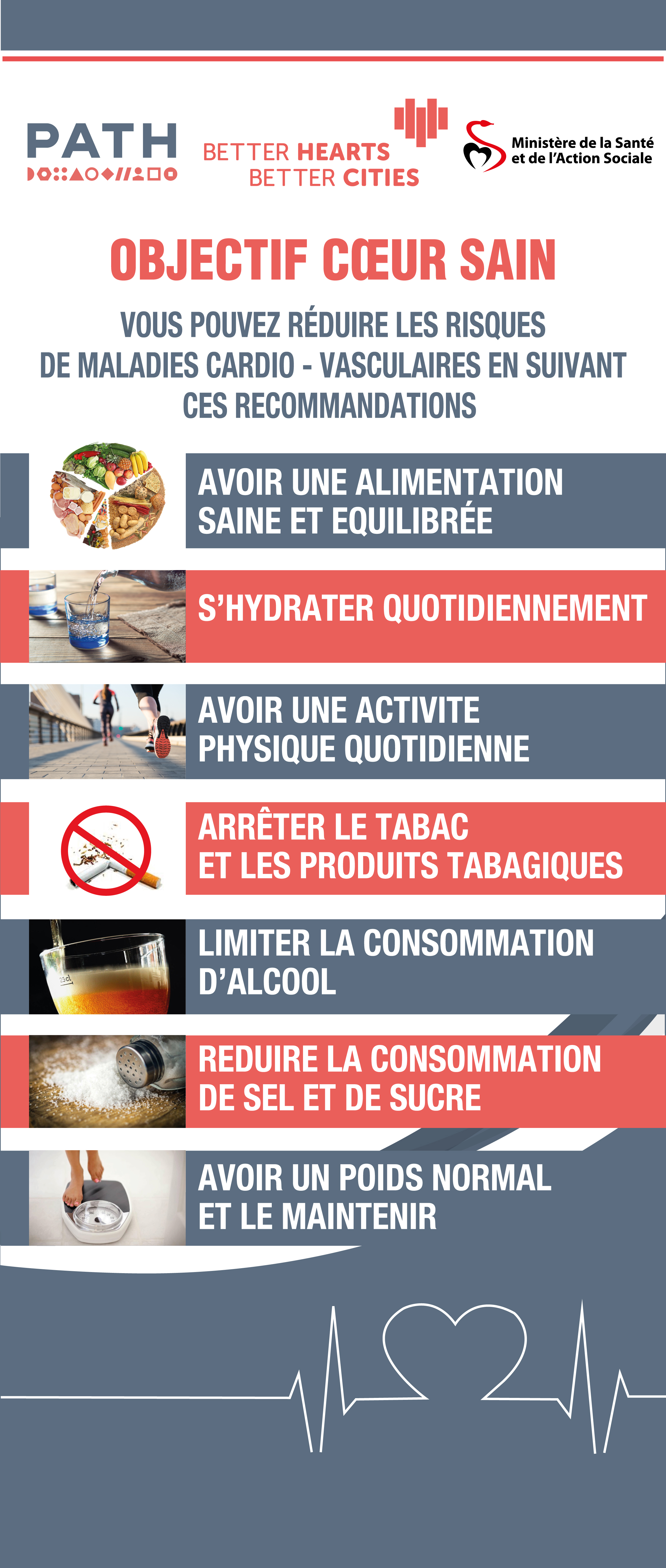

Supplement: Supplementary file 2 — Additional file 1. [file 12889_2021_11109_MOESM1_ESM.zip › Kakemono1.jpg]

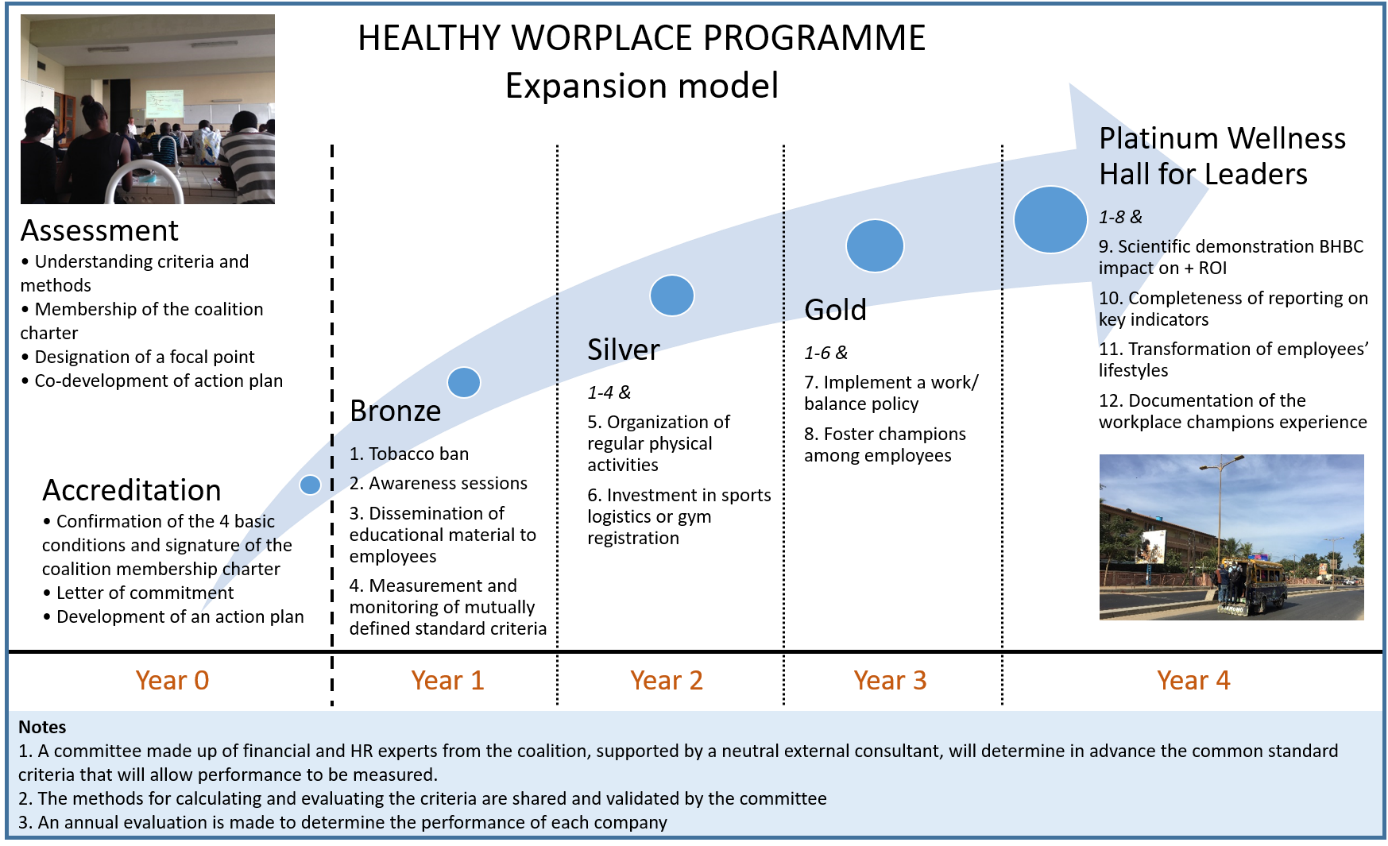

Supplement: Supplementary file 2 — Additional file 1. [file 12889_2021_11109_MOESM1_ESM.zip › Leaders Framework.docx]

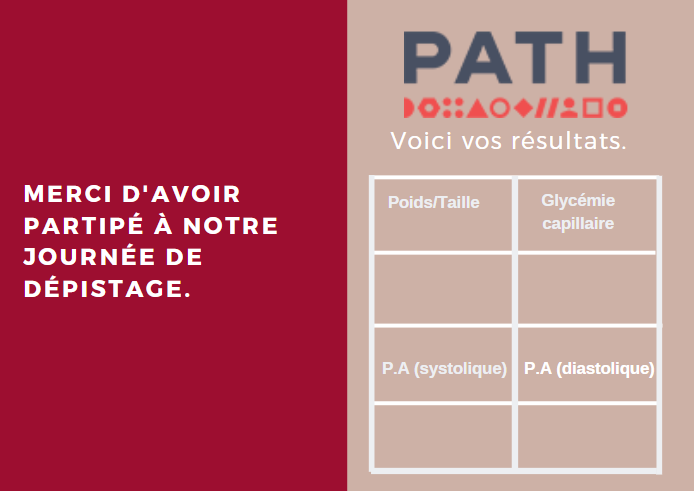

Supplement: Supplementary file 2 — Additional file 1. [file 12889_2021_11109_MOESM1_ESM.zip › Screening Cards Recto.PNG]

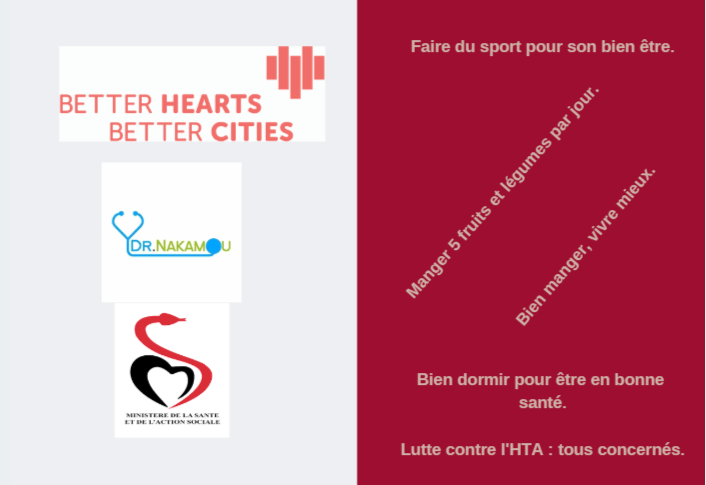

Supplement: Supplementary file 2 — Additional file 1. [file 12889_2021_11109_MOESM1_ESM.zip › Screening Cards Verso.PNG]
